# CBX7 suppresses urinary bladder cancer progression via modulating AKR1B10–ERK signaling

**DOI:** 10.1038/s41419-021-03819-0

**Published:** 2021-05-25

**Authors:** Zhengnan Huang, Yilin Yan, Zhen Zhu, Jiakuan Liu, Xiao He, Sumiya Dalangood, Meiqian Li, Mingyue Tan, Jinming Cai, Pengfei Tang, Ruimin Huang, Bing Shen, Jun Yan

**Affiliations:** 1 Department of Urology, Shanghai General Hospital, Shanghai Jiaotong University School of Medicine, 200080 Shanghai, China; 2 MOE Key Laboratory of Model Animals for Disease Study, Model Animal Research Center of Nanjing University, 210061 Nanjing, China; 3 Department of Laboratory Animal Science, Fudan University, 200032 Shanghai, China; 4 School of Chinese Materia Medica, Nanjing University of Chinese Medicine, 210023 Nanjing, China; 5 Shanghai Institute of Materia Medica, Chinese Academy of Sciences, 201203 Shanghai, China; 6 Department of Urology, Shuguang Hospital, Shanghai University of Traditional Chinese Medicine, 200021 Shanghai, China; 7 Department of Urology, Shanghai General Hospital Affiliated to Nanjing Medical University, 200080 Shanghai, China

**Keywords:** Tumour-suppressor proteins, Bladder cancer, Gene silencing, Bladder cancer

## Abstract

The chromobox (CBX) proteins mediate epigenetic gene silencing and have been implicated in the cancer development. By analyzing eight CBX family members in TCGA dataset, we found that chromobox 7 (CBX7) was the most strikingly downregulated CBX family member in urinary bladder cancer (UBC), as compared to normal tissues. Though dysregulation of CBX7 has been reported in multiple cancers, its specific role and clinical relevance in UBC remain unclear. Herein, we found that frequent downregulation of CBX7 in UBC specimens, which was due to its promoter hypermethylation, was correlated with poor prognosis. The ectopic expression of CBX7 suppressed UBC cell proliferation, migration, invasion, and cancer stemness, whereas CBX7 depletion promoted cancer cell aggressiveness. Importantly, CBX7 overexpression in UBC cells inhibited tumorigenicity, whereas CBX7 depletion promoted the tumor development, indicating its tumor-suppressive role in UBC. Using RNA-seq and chromosome immunoprecipitation (ChIP) assays, we identified aldo-keto reductase family 1 member 10 (AKR1B10) as a novel downstream target of CBX7, which was negatively modulated by CBX7 in a PRC1-dependent manner and involved in stimulating ERK signaling. Consistently, AKR1B10 overexpression induced cancer cell aggressiveness, whereas suppression of AKR1B10 by siRNA or its small molecular inhibitor, oleanolic acid, reversed the CBX7 deficiency-induced cellular effects. AKR1B10 overexpression was negatively associated with CBX7 downregulation and predicted poor clinical outcomes in UBC patients. Taken together, our results indicate that CBX7 functions as a tumor suppressor to downregulate AKR1B10 and further inactivates ERK signaling. This CBX7/AKR1B10/ERK signaling axis may provide a new therapeutic strategy against UBC.

## Introduction

Urinary bladder cancer (UBC) is one of the most common malignant tumors in the urinary system. The American Cancer Society predicts that male UBC in 2020 will be the fourth most common malignant tumor in the United States^1^. In China, its incidence ranks first among genitourinary system tumors with a yearly upward trend^2^. Despite various advancements in surgical and medical therapies, approximately half of UBC patients develop metastasis or recurrence within 2 years of diagnosis^3^. Thus, it is imperative to understand the molecular mechanisms underlying bladder carcinogenesis, in order to develop highly efficient treatment strategies.

The chromobox (CBX) family members function as epigenetic readers to regulate gene expression and have been implicated in various cancers^4–6^. Based on their structures and functions, this family can be categorized into two subgroups: heterochromatin protein 1 (HP1) and polycomb protein (Pc) subgroups. The HP1 subgroup contains CBX1, CBX3, and CBX5, which recognizes methylated histone H3K9 and contributes to heterochromatin formation and gene silencing^7^. Pc subgroup includes CBX2, CBX4, CBX6, CBX7, and CBX8, which are canonical components of polycomb repressive complex 1 (PRC1). Pc members recognize the trimethylation of histone H3 lysine 27 (H3K27me3), which is catalyzed by histone methyltransferase EZH2 in PRC2 complex, thereby driving the RING protein in PRC1 complex to ubiquitinate H2AK119. The interaction of PRC2 and PRC1 complex in chromatin also contributes to transcriptional silencing of target genes^8–11^. Among the CBX family members, CBX7 shows opposite functions in different cancer types. For example, CBX7 level was elevated in ovarian and gastric cancer, suggesting its oncogenic role in these tumor types^12,13^. In contrast, CBX7 plays an anticancer role and its reduction is related to the increased malignancy in thyroid, pancreatic, breast, and colon carcinomas^14–17^. Hence, its exact role in UBC awaits to elucidate.

Herein, we reported that the frequent downregulation of CBX7 mediated by promoter hypermethylation is associated with poor outcomes of UBC patients. We also performed functional studies to determine its role in UBC cell proliferation, migration/invasion, and cancer stemness. Next, we identified a downstream target gene of CBX7, which drives MAPK signaling pathway to facilitate cancer progression.

## Results

### The frequent downregulation of CBX7 predicts poor prognosis in UBC patients

**Fig. 1:**
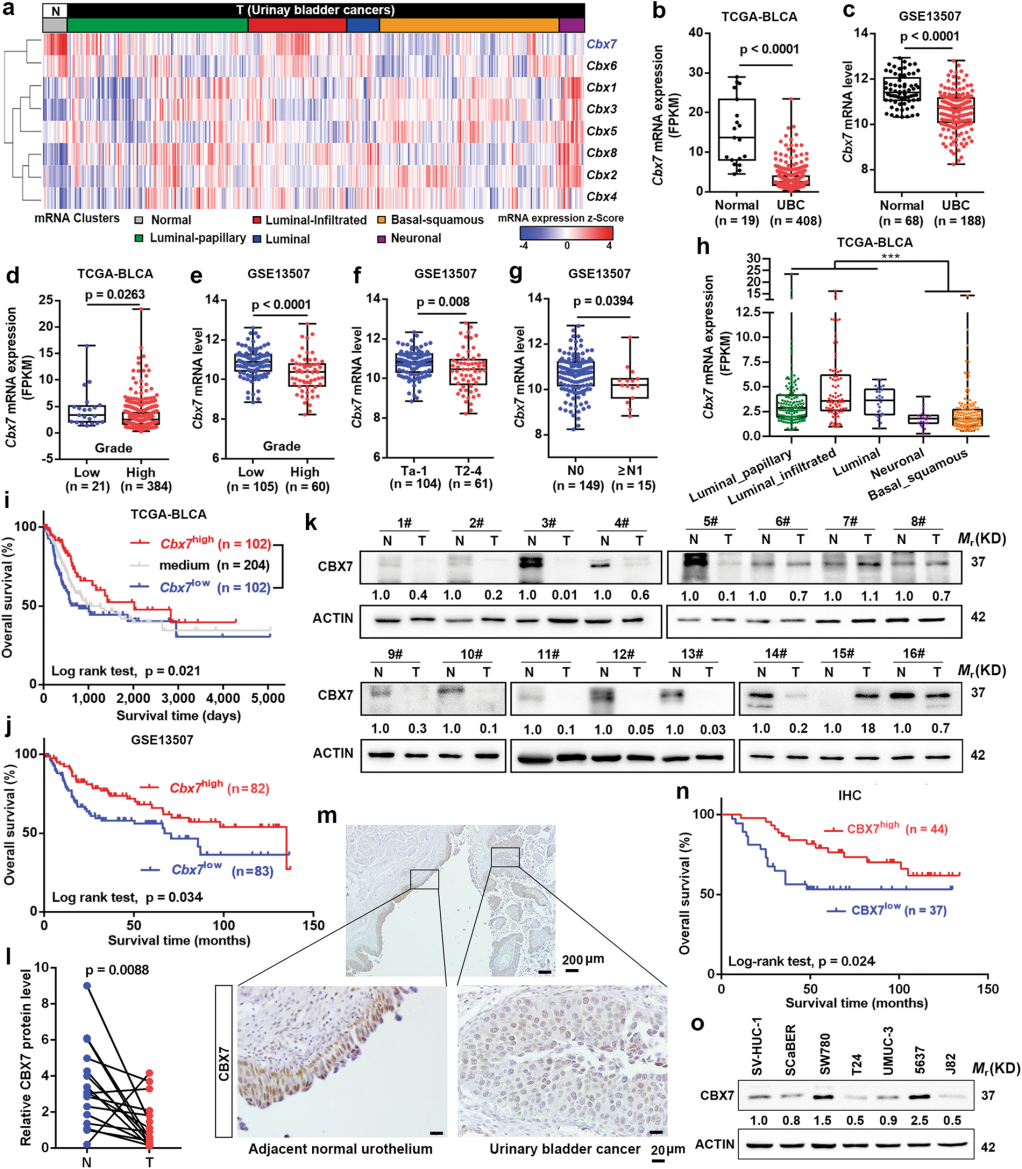
The frequent downregulation of CBX7 correlates with poor prognosis in UBC patients. **a** Hierarchical clustering illustrates the expression of CBX genes in TCGA-BLCA between UBC tissues and the adjacent normal tissues. **b**, **c** CBX7 mRNA expression between UBC tissues and the adjacent normal tissues in TCGA-BLCA and GSE13507. **d**–**g** The association of CBX7 mRNA expression with clinicopathologic features in TCGA-BLCA and GSE13507. **h** CBX7 mRNA expression in five UBC subtypes. **i**, **j** Kaplan–Meier plot of overall survival of UBC patients in TCGA-BLCA and GSE13507. **k**, **l** CBX7 protein levels in 16 paired UBC tissues (T) and their adjacent noncancerous tissues (N) were detected by western blotting. **m** IHC staining of CBX7 expression in a section containing UBC tissues and its tumor-adjacent noncancerous tissues. Scale bar, 200 μm (upper panel); 20 μm (lower panel). **n** Kaplan–Meier survival plot for CBX7 was constructed according to the IHC staining score. **o** Endogenous expression of CBX7 in a nonmalignant normal urothelial cell line and multiple human UBC cell lines by western blotting. Data are presented as means ± SD. ***p < 0.001.

To explore the potential prognostic value of different CBX members in UBC patients, we first surveyed the expression of CBXs between tumor and normal tissues in TCGA dataset. As shown in Fig. 1a, among all eight CBX family members, the CBX7 mRNA level was the most strikingly downregulated by 4.7-fold in UBC tissues compared to noncancerous tissues (*p* < 0.0001, Fig. 1b). Such reduced CBX7 level in UBC samples was further confirmed in another two independent datasets, GSE13507 (*p* < 0.0001, Fig. 1c) and GSE19915 (*p* < 0.0001, Fig. S1a). Moreover, CBX7 mRNA level was negatively correlated with clinicopathological features, such as grade (Fig. 1d, e), T stage (Fig. 1f and Fig. S1b), N stage (Fig. 1g), and M stage (Fig. S1c). Interestingly, we also found that downregulation of CBX7 was specifically in basal/squamous and neuronal subtypes, which were two UBC subtypes with poor prognosis (Fig. 1h). Consistently, UBC patients with low CBX7 mRNA level had shorter overall survival (OS) time than those with high CBX7 level in TCGA (*p* = 0.021, Fig. 1i), GSE13507 (*p* = 0.034, Fig. 1j), and GSE19915 (*p* = 0.0319, Fig. S1d) datasets.

To further validate its downregulation in UBC samples, we performed western blotting and observed a significant reduction of CBX7 protein level in 14 out of 16 UBC samples (*p* = 0.0088, Fig. 1k, l). Immunohistochemistry (IHC) staining showed that CBX7 notably expressed in the nuclei of normal urothelial cells, but weakly positively stained in the UBC cells (Fig. 1m). The representative images for different staining intensities of CBX7 were shown in Fig. S1e. CBX7 protein level was remarkably correlated with T stage (*p* < 0.001) and tumor grade (*p* = 0.002), but not related with other features, such as age and gender (Table S1). Kaplan–Meier plot and Cox regression analysis supported the notion that lower CBX7 protein level is an independent prognostic marker in UBC patients (*p* = 0.024, Fig. 1n and Table S2). In line with the aforementioned findings, CBX7 protein expression level was lower in most UBC cell lines as compared to nonmalignant urothelial cell line SV-HUC-1 (Fig. 1o). In summary, we concluded that CBX7 was frequently reduced in UBC, and its downregulation was significantly associated with poor prognosis in UBC patients.

### CBX7 inhibits UBC cells growth through the induction of cell cycle arrest

**Fig. 2:**
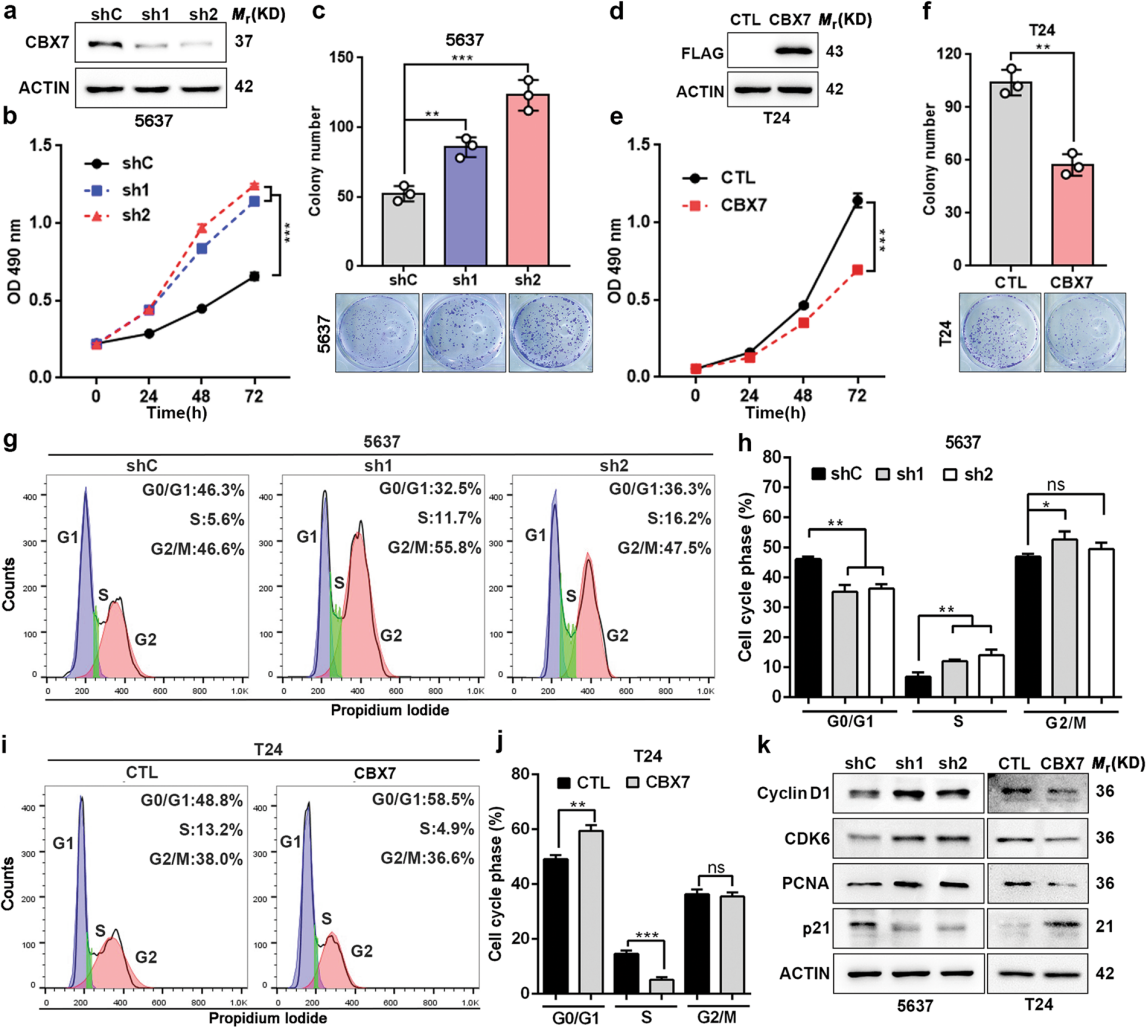
CBX7 inhibits UBC cells proliferation through the induction of cell cycle arrest. **a** Knockdown of CBX7 in 5637 cell was confirmed by western blotting. **b**, **c** Effects of CBX7 knockdown on cell viability and colony formation **d** Ectopic expression of CBX7 in T24 cell was confirmed by western blotting. **e**, **f** Effects of CBX7 overexpression on cell viability and colony formation. **g**–**j** Effects of CBX7 on cell cycle were determined by flow cytometry analysis. **k** Certain cell cycle-related regulators were analyzed by western blotting. Data are presented as means ± SD from three independent replicates. *p < 0.05, **p < 0.01, ***p < 0.001, ns nonsignificant.

The frequent downregulation of CBX7 in UBC tissues suggested that CBX7 may function as a tumor suppressor. To confirm this hypothesis, we first explored the effects of CBX7 on cell proliferation. As shown in Fig. 2a–c, knockdown of CBX7 significantly promoted 5637 cell proliferation and increased colony numbers. Congruently, ectopic expression of CBX7 markedly inhibited T24 and UMUC-3 cells viability and colony formation ability (Fig. 2d–f and Fig. S2a–c). Flow cytometry analysis revealed that CBX7 depletion significantly decreased G1-phase and increased S-phase in 5637 cells (Fig. 2g, h), whereas overexpression of CBX7 elevated G1-phase, but reduced S-phase in T24 (Fig. 2i, j) and UMUC-3 cells (Fig. S2d, e). In line with this, knockdown of CBX7 upregulated the protein expression of the G1–S transition promoters (cyclin D1 and CDK6), as well as cell proliferation marker (PCNA), but downregulated the G1 gatekeeper p21, whereas ectopic CBX7 downregulated cyclin D1, CDK6, and PCNA, but upregulated p21 (Fig. 2k and Fig. S2a). These results indicated that CBX7 inhibited UBC cell proliferation.

### CBX7 suppresses UBC cell invasion and migration

**Fig. 3:**
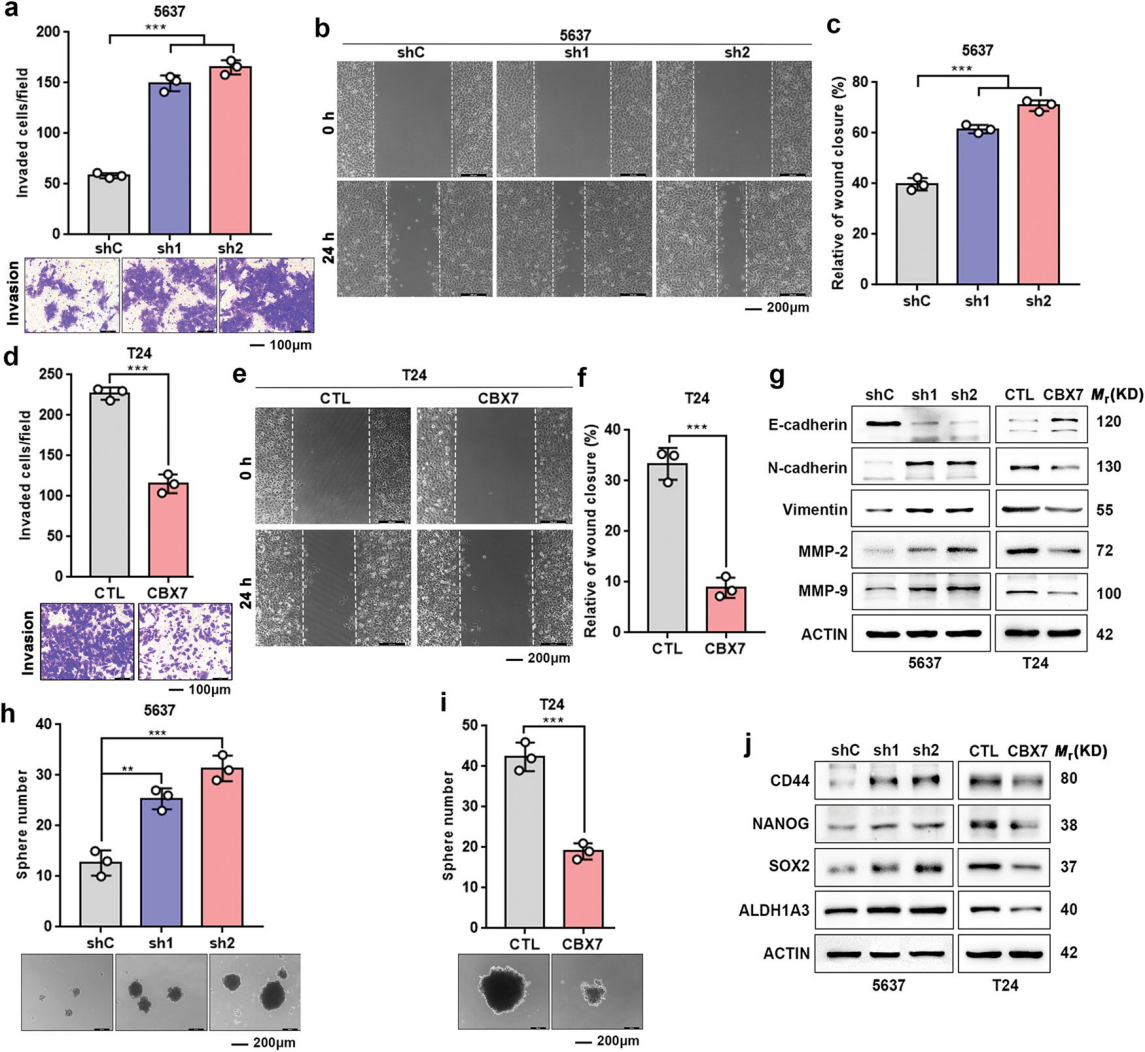
CBX7 suppresses UBC cells invasion, migration, and stemness. **a**–**c** Effects of CBX7 knockdown on cell invasion (**a**) and migration (**b**, **c**). **d**–**f** Effects of CBX7 overexpression on cell invasion and migration. **g** Epithelial and mesenchymal transition markers, and matrix metalloproteinases were detected by western blotting. **h**, **i** Effects of CBX7 on the size and number of spheres. **j** The levels of core stem cell genes were detected by western blotting. Scale bar, 100 μm (**a**, **d**); 200 μm (**b**, **e**, **h**, **i**). Data are presented as means ± SD from three independent replicates. **p < 0.01, ***p < 0.001.

We subsequently explored the effects of CBX7 on cell invasion and migration capacities using transwell and wound healing assays. Cancer cell invasiveness was significantly enhanced when CBX7 was knocked down in 5637 cells (Fig. 3a), but overexpression of CBX7 suppressed T24 and UMUC-3 cell invasiveness (Fig. 3d and Fig. S3a). Wound healing also validated that CBX7 knockdown facilitated cell movement (Fig. 3b, c). On the contrary, cells overexpressing CBX7 filled the gap slower than control cells (Fig. 3e, f and Fig. S3b, c). Consistently, western blotting showed that CBX7 regulated the epithelial–mesenchymal transition (EMT) through the upregulation of E-cadherin and the downregulation of N-cadherin, Vimentin, and matrix metalloproteinases (MMP-2 and MMP-9; Fig. 3g and Fig. S3d). In contrast, knockdown of CBX7 in 5637 cells repressed E-cadherin, but elevated the expression levels of N-cadherin, Vimentin, MMP-2, and MMP-9 (Fig. 3g). Collectively, these data implied that CBX7 suppresses UBC cells invasion and migration capacities.

### CBX7 impairs UBC cell stemness

To examine whether CBX7 could influence UBC cell stemness, sphere formation experiments were performed. The results showed that the sphere formation ability was enhanced in CBX7-depleted 5637 cells (Fig. 3h), but impaired by CBX7 overexpression in T24 and UMUC-3 cells (Fig. 3i and Fig. S3e). Consistently, the expression of stem cell markers, such as CD44, SOX2, NONOG, and ALDH1A3, were induced by CBX7 depletion, but reduced by CBX7 overexpression (Fig. 3j and Fig. S3f). Collectively, these findings indicated that CBX7 inhibits UBC cell stemness.

### Promoter methylation leads to the downregulation of CBX7 in UBC cells

**Fig. 4:**
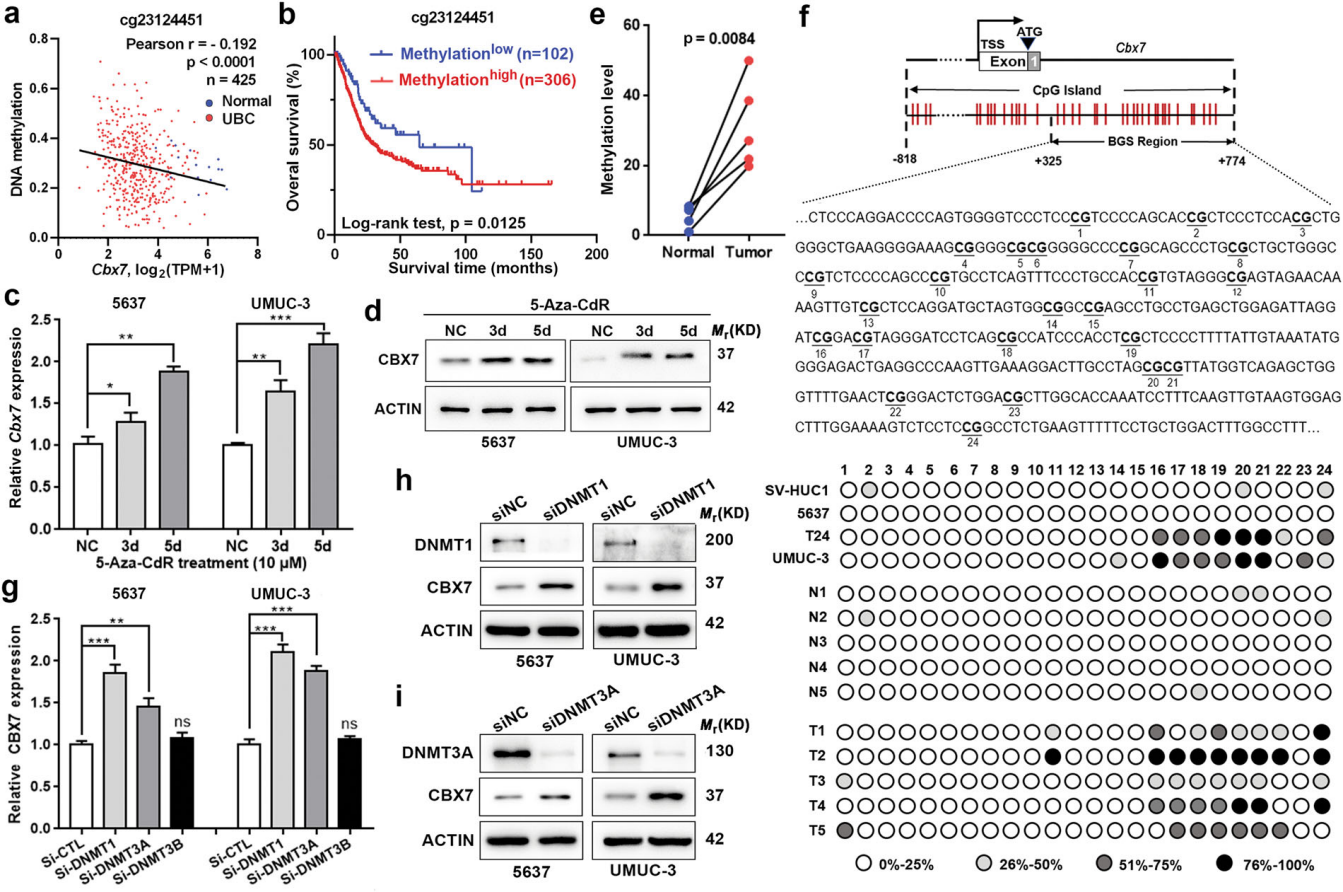
CBX7 is inactivated by promoter hypermethylation mediated by DNMT1 and DNMT3A in UBC. **a** Pearson correlation analysis showed a significant negative correlation between CBX7 methylation level and mRNA expression from TCGA dataset. **b** Kaplan–Meier survival plot was constructed according to the methylation level. **c**, **d** mRNA and protein levels of CBX7 were restored in UBC cells after treatment with 5-Aza-CdR at 10 μM for the indicated times. **e**, **f** Bisulfite genomic sequencing (BGS) analysis of CBX7 CpG island region. Methylation status of CBX7 was observed in UBC cell lines and paired UBC tissues (T) and their adjacent noncancerous tissues (N). **g** CBX7 mRNA levels were examined in DNMT1, DNMT3A, or DNMT3B-silenced 5637 and UMUC-3 cells by qRT-PCR. **h**, **i** Western blotting results showed that knockdown of DNMT1 and DNMT3A in 5637 and UMUC-3 cells significantly elevated the level of CBX7. Data are presented as means ± SD from three independent replicates. *p < 0.05, **p < 0.01, ***p < 0.001, ns nonsignificant.

Due to its frequent downregulation in UBC, we speculated that CBX7 might be regulated by promoter methylation, a major mechanism contributing to the inactivation of tumor suppressor genes^18^. By comparing the CBX7 mRNA and its methylation level in TCGA database, we found that CBX7 mRNA level was negatively associated with its promoter methylation (*n* = 425, probe cg23124451, *p* < 0.0001, Fig. 4a), indicating promoter methylation exerted a crucial regulatory effect on CBX7 expression in UBC. Interestingly, CBX7 promoter hypermethylation status predicts poor clinical outcomes (*p* = 0.0125, Fig. 4b). Furthermore, when two UBC cell lines, 5637 and UMUC-3, were treated with DNA demethylation agent 5-Aza-CdR at 10 μM for 3–5 days, we observed the elevation of CBX7 expression at mRNA and protein levels (Fig. 4c, d). Next, bisulfite genomic sequencing (BGS) analysis was performed to evaluate the methylation status of CBX7 in the CpG island of the promoter region covering 24 CpG sites of the CBX7 gene. Rare methylation was detected in high CBX7-expressing SV-HUC-1 and 5637 cells, whereas relatively dense methylation was detected in low CBX7-expressing UMUC-3 and T24 cells (Fig. 4f). At last, the CXB7 promoter methylation was significantly higher in UBC tissues than their non-tumorous counterparts in five paired UBC tissues and adjacent normal tissues (Fig. 4e, f).

Since DNA methyltransferase (DNMT) family members, DNMT1, DNMT3A, and DNMT3B, play crucial roles in maintaining DNA methylation, we examined the CBX7 expression level in DNMT1, DNMT3A, or DNMT3B-depleted 5637 and UMUC-3 cells. The results showed that knockdowns of DNMT1 and DNMT3A, but not DNMT3B, significantly elevated CBX7 at both mRNA and protein levels, respectively (Fig. 4g–i). Altogether, the downregulation of CBX7 is due to its promoter hypermethylation, mediated by DNMT1 and DNMT3A in UBC.

### CBX7 suppresses the expression of AKR1B10 in a PRC1-dependent manner and thereby inhibits ERK signaling

**Fig. 5:**
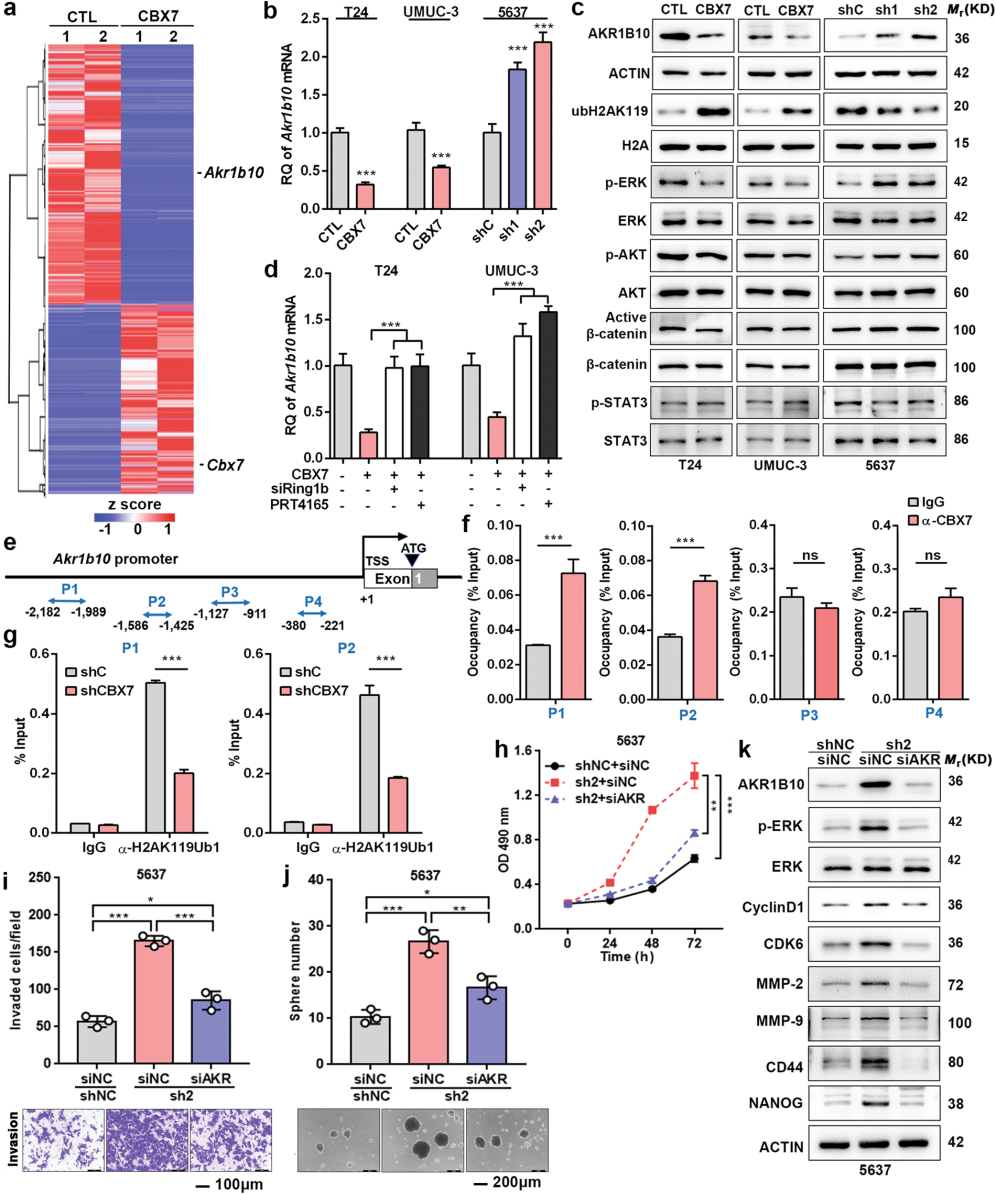
CBX7 suppresses UBC progression via suppression of AKR1B10 in a PRC1-dependent manner and thereby inhibits ERK signaling. **a** RNA transcriptome sequencing analysis was performed to examine the altered expression of genes by CBX7 overexpression in T24 cells. **b** AKR1B10 mRNA expression level upon CBX7 overexpression or knockdown. **c** Western blotting was performed to detect genes expression upon CBX7 overexpression or knockdown. **d** Cells with CBX7 overexpression were treated with Ring1b siRNA or RING inhibitor PRT-4165 for 24 h. The mRNA expression of AKR1B10 was determined by qRT-PCR. **e** A schematic representation of the AKR1B10 promoter studied by ChIP analysis. **f** Quantitative ChIP analysis of CBX7 in AKR1B10 in 5637 cells. **g** Quantitative ChIP analysis of H2AK119ub1 in AKR1B10 in 5637 CBX7-silenced cells. **h**–**j** CBX7 knockdown increased cell viability, invasive ability, and the size and number of spheres in 5637 cells, whereas this effect was partly abolished upon AKR1B10 suppression (AKR means AKR1B10). Scale bar, 100 μm (**i**); 200 μm (**j**). **k** Western blotting was used to examine genes expression in CBX7 knockdown 5637 cells transiently transfected with siNC and siAKR1B10. Data are presented as means ± SD from three independent replicates. *p < 0.05, **p < 0.01, ***p < 0.001.

To further dissect the molecular mechanism of CBX7-mediated anticancer effects, transcriptional profiling by RNA-seq was conducted with CBX7-overexpressing T24 and control cells (Fig. 5a). Among the differential candidate genes, we examined their consistent changes in both CBX7-overexpressing and CBX7-silencing cells by quantitative reverse transcription-PCR (qRT-PCR). Finally, aldo-keto reductase family 1 member 10 (AKR1B10) was the gene we detected that showed a strong negative correlation with CBX7 expression (Fig. S4a–c and Fig. 5b). Western blotting also validated that AKR1B10 was downregulated upon CBX7-overexpressing in T24 and UMUC-3 cells, while upregulated upon CBX7-silencing in 5637 cells (Fig. 5c). To determine whether CBX7 regulates AKR1B10 in a PRC1-dependent manner, we chose to suppress Ring1b, a histone H2A ubiquitin ligase in the PRC1 complex. The modulation of AKR1B10 by CBX7 was abolished by either treatment of Ring1b siRNA or its small molecular inhibitor, PRT-4165 (Fig. 5d). Furthermore, western blotting showed the expression of ubiquitinated H2AK119 was elevated in T24 and UMUC-3 cells overexpressing CBX7, while reduced in 5637 cells with CBX7 knockdown (Fig. 5c). Next, ChIP assays were carried out to examine whether CBX7 could directly bind to the promoter of AKR1B10. Four primers were designed covering the promoter region of AKR1B10 locus (P1–P4, nt −2182 to −221, Fig. 5e). As shown in Fig. 5f, ChIP data revealed that CBX7 was recruited mainly to the P1 and P2 promoter regions, but not P3 and P4 regions. Meanwhile, the level of mono-ubiquitinated H2AK119 was significantly reduced in the P1 and P2 promoter regions of the *Akr1b10* gene, once CBX7 was silenced (Fig. 5g). Taken together, these data indicated that CBX7 modulated the AKR1B10 expression in a PRC1-dependent manner.

Next, to characterize the role of AKR1B10 as the downstream target of CBX7, AKR1B10 was depleted by siRNA in CBX7-silencing cells (Fig. 5k). We observed that the suppression of AKR1B10 partly negated the ability of cell growth, invasion and sphere formation induced by CBX7-silencing (Fig. 5h–j).

To further investigate the downstream signaling pathways of CBX7–AKR1B10 axis, western blotting was used to detect the protein levels of active β-catenin, p-AKT, p-STAT3, and p-ERK, the key molecules in the classic signaling pathways, which have been reported to participate in diverse cellular processes such as proliferation, survival, and differentiation in cancer cells. We found the level of p-ERK altered most strikingly and consistently in cells overexpressing or knocking down CBX7 (Fig. 5c). The activated ERK signaling could directly or indirectly modulate cell cycle-related proteins, such as CDKs and cyclins controlling G1–S transition phase and pro-metastatic factors, such as EMT markers and MMPs, as well as some cancer stem cell-related genes, such as CD44 and NANOG^19–23^. Therefore, the levels of ERK, p-ERK; G1–S transition promoter, cyclin D1, and CDK6; matrix metalloproteinases, MMP-2 and MMP-9; and stem cell markers, CD44 and NANOG were determined by western blotting in CBX7-silencing 5637 cells with or without AKR1B10 repression. As shown in Fig. 5k, these genes were upregulated in CBX7 knockdown cells, whereas their expression levels were reduced in AKR1B10 knockdown cells. Altogether, these data indicated that CBX7 deficiency triggers the activation of AKR1B10-mediated ERK signaling and related downstream targets, leading to the UBC aggressiveness.

### AKR1B10 promotes UBC cells progression via stimulating ERK signaling

**Fig. 6:**
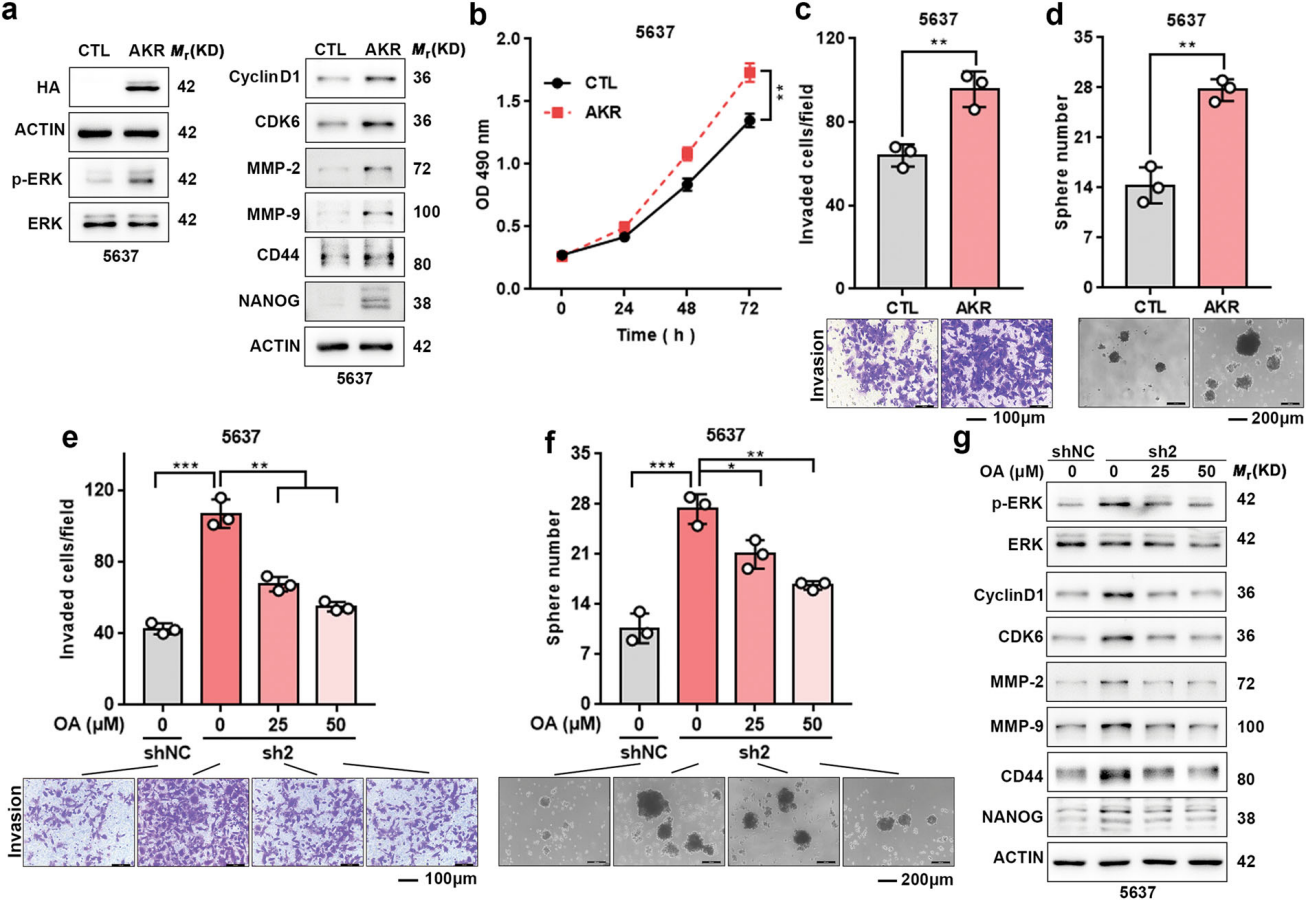
AKR1B10 promotes UBC progression and oleanolic acid inhibits UBC progression via suppressing the AKR1B10–ERK signaling. **a** Western blotting was performed to examine genes expression in AKR1B10 overexpression 5637 cells. **b**–**d** Effects of AKR1B10 overexpression on cell viability, invasion, and sphere formation in 5637 cells. **e**, **f** CBX7 knockdown increased invasive ability and the size and number of spheres in 5637 cells, whereas this effect was abolished upon AKR1B10 inhibition by OA at dose of 25 and 50 μM after treated 72 h. **g** Western blotting was performed to detect genes expression in CBX7 knockdown 5637 cells treated with OA at dose of 25 and 50 μM after treated 72 h. Scale bar, 100 μm (**c**, **e**); 200 μm (**e**, **f**). Data are presented as means ± SD from three independent replicates. *p < 0.05, **p < 0.01, ***p < 0.001.

To further confirm whether AKR1B10 promotes UBC aggressiveness, we examined the effect of AKR1B10 on the cell growth, invasion, and stemness by overexpressing AKR1B10 in 5637 cells. Ectopic expression of AKR1B10, confirmed by western blotting (Fig. 6a), markedly increased the ability of cell growth, invasion, and sphere formation (Fig. 6b–d), as well as stimulating the ERK signaling and its downstream molecules expression (Fig. 6a). These results further confirmed the conclusion that AKR1B10 played a tumor promoting role to contribute the progression of UBC by activating ERK signaling.

### Oleanolic acid, an AKR1B10 inhibitor, inhibits UBC progression via suppressing the AKR1B10–ERK signaling

Next, we explored the potential therapeutic target of AKR1B10 in UBC. It has been reported that oleanolic acid (OA) is a potent AKR1B10 inhibitor^24^, which can significantly inhibit the enzyme activity of AKR1B10 and thereby suppress cell growth in pancreatic cancer^25^. Therefore, we investigated whether the pharmacological inhibition of AKR1B10 by OA could exert similar effect to that of AKR1B10 knockdown by using siRNA. By transwell and sphere formation assays, we found that OA at 25 and 50 μM significantly reduced the invaded cells (Fig. 6e), and inhibited the number and size of spheres (Fig. 6f). Consistently, treatment with OA suppressed the ERK signaling, as indicated by the reduced expression of its downstream targets, including cyclin D1, CDK6, MMP-2, MMP-9, CD44, and NANOG (Fig. 6g). These findings indicated that pharmacological inhibition of AKR1B10 by OA had negative effects on UBC aggressiveness via suppressing the AKR1B10–ERK pathway.

### CBX7 inhibits tumorigenicity in vivo

**Fig. 7:**
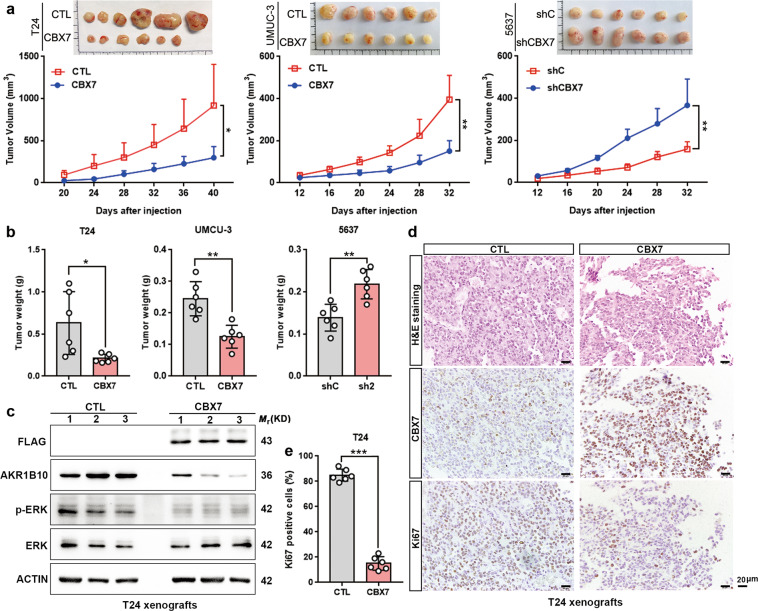
CBX7 inhibits tumorigenicity in vivo. **a**, **b** Tumor growth curves and tumor weights of xenografts were represented. **c** CBX7, AKR1B10, p-ERK, and ERK expression in the xenografts of T24-CBX7 were determined by western blotting. **d**, **e** H&E staining was used to monitor UBC cells morphology, IHC staining for CBX7 substantiated ectopic CBX7 expression, and Ki-67 staining revealed a significant reduction of cell proliferation in T24 xenografts expressing CBX7 by counting the proportion of Ki-67-positive cells. Scale bar, 20 μm. Data are presented as means ± SD of six mice from each group. *p < 0.05, **p < 0.01, ***p < 0.001.

Next, we estimated the role of CBX7 on tumorigenicity in vivo. Cells with CBX7 overexpression or depletion were injected into the flanks of nude mice, respectively. As a result, the tumor volumes and sizes were dramatically smaller in the CBX7-overexpressed T24 and UMUC-3 cells (*p* < 0.05; Fig. 7a). Conversely, tumors formed by CBX7-silenced 5637 cells were significantly larger than those in the control group (*p* < 0.05; Fig. 7a). Furthermore, the tumors were much lighter in the CBX7-overexpression groups, but much heavier in the CBX7-depletion groups, compared to the corresponding control groups (*p* < 0.05; Fig. 7b). Western blotting confirmed that AKR1B10 and p-ERK level were repressed upon CBX7 overexpression in vivo (Fig. 7c). Histological staining and Ki-67 staining clearly demonstrated that the cell proliferation index was decreased in T24 and UMUC-3 xenografts overexpressing CBX7, but increased in 5637 xenografts with the depletion of CBX7, compared to the control groups (Fig. 7d, e and Fig. S5a–d). In summary, these data supported the tumor-suppressive function of CBX7.

### The dysregulation of CBX7–AKR1B10 axis is associated with poor clinical outcome

**Fig. 8:**
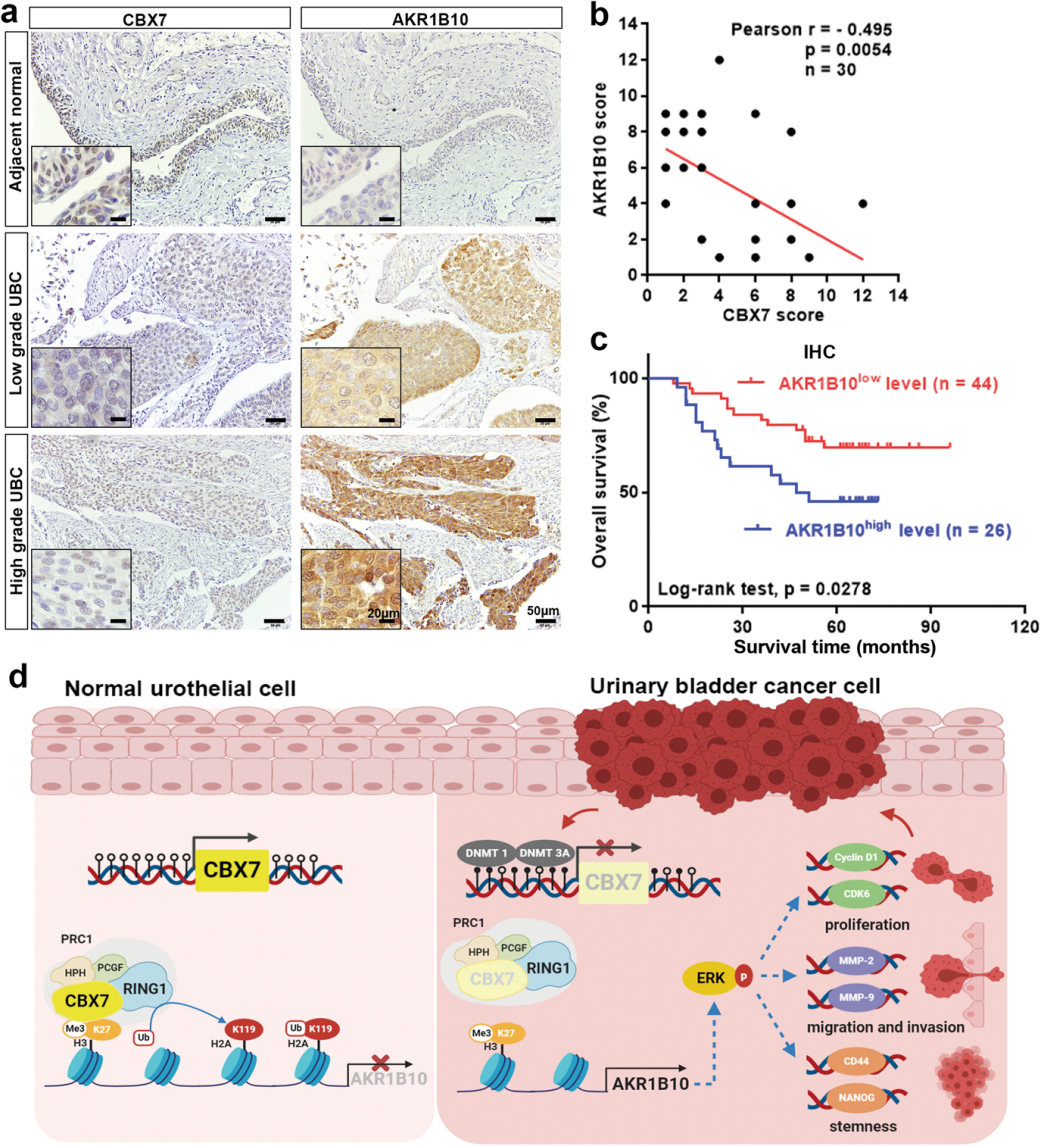
The dysregulation of CBX7–AKR1B10 axis is associated with poor clinical outcome. **a** Representative IHC staining images of the localization and expression level of CBX7 and AKR1B10 in adjacent normal tissues and UBC tissues were displayed. Scale bar, 50 μm; inset, 20 μm. **b** Pearson correlation analysis showed a significant negative correlation between CBX7 and AKR1B10 expression. **c** Kaplan–Meier survival plot for AKR1B10 was constructed according to the IHC staining score. **d** A working model of CBX7/AKR1B10/ERK axis in UBC progression.

To further explore the clinical correlation between CBX7 and AKR1B10 in UBC, the same cohort of 30 UBC patients were enrolled to perform IHC analysis. The results showed that in the adjacent normal urinary epithelial tissues, the staining of CBX7 was intense, but rare of AKR1B10. While in tumor tissues, the higher grade of tumor is, the lower expression level of CBX7, but the higher expression level of AKR1B10 were detected (Fig. 8a). Interestingly, CBX7 expression was negatively correlated with AKR1B10 expression in UBC samples (*n* = 30, *p* = 0.0054, Fig. 8b). In addition, patients with high AKR1B10 level predicted worse prognosis than those with low AKR1B10 level (*p* = 0.0278, Fig. 8c). Further analysis revealed that AKR1B10 protein level was strongly associated with tumor grade (*p* = 0.005, Table S3; *p* = 0.0006, Fig. S4d). The representative images for different staining intensities of AKR1B10 were shown in Fig. S4e.

Taken together, CBX7 is downregulated in UBC due to the promoter methylation mediated by DNMT1 and DNMT3A, eliminating the CBX7-mediated inhibitory effect of AKR1B10, thereby leading to the upregulation of AKR1B10 and activation of ERK signaling (Fig. 8d).

## Discussion

As the important components of the epigenetic regulatory complexes, CBX family proteins are involved in the development of multiple cancers, including UBC^6,7,26,27^. In this study, we found that CBX7 is the most remarkably downregulated gene among CBX family members in UBC tissues, as compared to normal tissues in TCGA dataset. Consistently, we demonstrated that CBX7 was generally expressed in normal human urothelial tissues, but frequently silenced in UBC cell lines and primary tumor tissues due to promoter hypermethylation. Consistent with our findings, hypermethylated CpG island of CBX7 promoter was also observed in glioblastoma^28^. Moreover, we validated that DNMT1 and DNMT3A were responsible for the aberrant promoter methylation of CBX7. Relevantly, our previous study revealed that TET1, a pivotal regulator of DNA demethylation contrary to DNMTs, its downregulation promoted UBC progression^29^. Recent study provided a comprehensive review about DNMT inhibitors serving as a therapeutic target for the treatment of UBC^30^. Collectively, these findings indicated that DNA methylation was a promising target for UBC therapy.

CBX7 silencing in UBCs suggested that CBX7 might act as a tumor suppressor, and its downregulation probably contributes to the development and progression of UBC. To confirm our hypothesis, a series of functional experiments in vitro and in vivo were carried out. The ectopic overexpression of CBX7 inhibited the cell growth in UBC cell lines in vitro and tumorigenesis in vivo; while knockdown of CBX7 facilitated the cell growth. Meanwhile, CBX7 suppressed the cell migration and invasion abilities and impaired cell stemness. Taken together, these results strongly indicated that CBX7 functioned as a novel tumor suppressor in UBC.

To elucidate the underlying mechanisms of CBX7-mediated anticancer effects, AKR1B10 was identified as a potential novel downstream target of CBX7 through transcriptome sequencing analysis. The canonical manner for CBXs in PRC1 complex to exhibit function is as a transcriptional suppressor of downstream targets, such as miR-137 and INK4a/ARF^31,32^. Consistently, our data demonstrated that CBX7 functioned as a transcriptional inhibitor in a PRC1-dependent manner to suppress the transcription of AKR1B10. In addition, we found CBX7 was negatively correlated with AKR1B10 in the cohort of clinical UBC samples, further suggesting that AKR1B10 served as a downstream target of CBX7. Then, we demonstrated that suppression of AKR1B10 by siRNA could reverse the tumor promoting effect induced by CBX7 knockdown on cell proliferation, invasion, and stemness. However, these effects were not fully rescued by AKR1B10 depletion, indicating other tumor suppression mechanisms mediated by CBX7. For example, CBX7 positively regulated E-cadherin expression by inhibiting HDAC2 and PRMT1 activity on the E-cadherin promoter, and thereby suppressed cancer progression^33,34^.

In the gain-of-function assays, overexpression of AKR1B10 facilitated UBC progression, and patients with high AKR1B10 level predicted worse clinical outcome than those with low AKR1B10 level. Recently, AKR1B10 was reported to be overexpressed in a wild range of cancers, including hepatocellular carcinoma, breast cancer, pancreatic carcinoma, and lung cancer^35–38^. Besides, AKR1B10 expression was also reported to upregulate in carboplatin–gemcitabine combination chemotherapy cancer patients^39^. Taken together, these data suggested AKR1B10 might function as an oncogene contributing to the cancer progression and clinical poor outcome. Meanwhile, AKR1B10–ERK signaling had been reported to be involved in the tumor aggressiveness in breast cancer and lung adenocarcinoma^40,41^. In the present study. we also substantiated that AKR1B10 could stimulate the ERK signaling in UBC. However, the specific mechanism contributing to the AKR1B10-induced activation of ERK signaling in UBC remains unclear and needs to further explore.

Previous study had demonstrated that OA was the most potent AKR1B10 competitive inhibitor, which was used in pancreatic cancer to inhibit the AKR1B10 activity, and thereby suppressed cell growth in vitro and in vivo^24,25^. In line with the above findings, our data showed suppression of AKR1B10 by OA could also rescue the tumor promoting effect of CBX7 knockdown on cell proliferation, invasion, and stemness, implying OA would be a promising pharmacological inhibition for targeting AKR1B10 in UBC. Taken together, suppression of AKR1B10 by siRNA approach or inhibitor in CBX7-silencing UBC cells could contribute to a significant reduction of cell proliferation, invasion, and sphere formation.

In summary, our data demonstrated that CBX7 expression was silenced in UBC due to promoter hypermethylation and downregulation of CBX7 was correlated with unfavorable clinical outcome. We also demonstrated for the first time that CBX7 acted as a tumor suppressor in UBC through the inhibition of cell proliferation, invasion, and stemness, via transcriptionally suppressing the expression of AKR1B10–ERK signaling. This newly identified CBX7/AKR1B10/ERK axis may provide new therapeutic strategies against UBC.

## Materials and methods

### Cell lines and chemicals

The normal urothelial cell (SV-HUC-1) and UBC cell lines (J82, T24, 5637, SW780, UMUC-3, and SCaBER) were purchased from the Cell Bank of Type Culture Collection of Chinese Academy of Sciences (Shanghai, China). All cell lines were recently authenticated and tested for mycoplasma contamination. All cells were cultured in RPMI 1640 medium (Life Technologies) supplemented with 10% FBS (Hyclone) at 37 °C with 5% CO_2_. Chemicals and reagents used in this study were listed in Table S4.

### Bioinformatics analysis

Gene expression and clinicopathologic information of Gene Expression Omnibus (GEO) databases are publicly available. Gene expression, DNA methylation, and clinical date of The Cancer Genome Atlas Urothelial Bladder Carcinoma (TCGA-BLCA) were retrieved from Genomic Data Commons Data Portal. The mRNA subtypes of TCGA-BLCA (*n* = 408) had been previously developed^42^. The gene mRNA expression in each subtype were described, as previous reported^43^. The beta value of CpG sites were collected to evaluate the association of CBX7 mRNA expression in promoter and gene body region. The cg23124451 was selected to show the correlation coefficient of CBX7 expression and methylation.

### Clinical samples

Twenty-one pairs of fresh UBC tissues and adjacent non-tumorous tissues were acquired from the patients at the time of surgery from the Shanghai General Hospital. A total of paraffin-embedded UBC tissues and clinical information were also collected. All patients signed an informed consent form, and the study protocol acquired official approval by the ethics committees of the Shanghai General Hospital.

### RNA isolation and quantitative reverse transcription-PCR

TRIzol reagent (TaKaRa, Dalian, China) was used to isolate the total RNA of cultured cells. Prime-Script RT-PCR kit (TaKaRa) was applied to reverse transcribe RNA into cDNA. The expression levels of genes were measured in an ABI 7500 StepOne Plus Real-Time PCR instrument (Applied Biosystems). The specific primer sequences used were listed in Table S5.

### Western blotting

RIPA buffer containing phosphatase and protease inhibitor was used to extract total protein in tissues or cells. For the detection of histone H2A ubiquitination levels, cells were lysed in SDS lysis buffer. Subsequently, proteins were separated by SDS–PAGE and transferred onto the PVDF membrane. After blocking in 5% nonfat milk with PBST, the membranes were incubated with primary antibodies. The corresponding secondary antibodies were used after the membranes were washed in PBST three times. ECL substrate kit (Tanon Science and Technology, Shanghai, China) was used to visualize the western blots. Antibodies used were listed in Table S6. The whole untrimmed western blotting images were presented in Fig. S6.

### Stable cell line establishment

For CBX7 knockdown, two shRNA plasmids targeting CBX7 were constructed using the lentiviral pLKO.1 backbone with puromycin resistance. The shRNA sequences were listed in Table S5. For gene overexpression, the full-length CBX7 and AKR1B10 were respectively cloned into pCDH-3xFLAG vector plasmids and pCMV-3xHA vector plasmids.

### Cell proliferation assay

MTT (3-(4,5-dimethylthiazol-2-yl)-2,5-diphenyltetrazolium bromide) assay was carried out to evaluate the cell growth. A total of 2000 cells/well were seeded to the 96-well plate until the cells adhered to the bottom, which was identified as 0 timepoint. A total of 10 μl MTT was added to each well and removed after incubating for 3 h at 37 °C at 24, 48, and 72 h. Then, 100 μl dimethyl sulfoxide was added into each well and incubated at 37 °C for 15 min. Absorbance at 490 nm was examined using a microplate reader (BioTek Instruments, Winooski, VT).

### Colony formation assay

A total of 2000 cells/well were seeded in six-well plates. After 14 days, cells were fixed in formaldehyde and stained with 0.2% crystal violet. Only colonies with >50 cells were counted.

### Cell cycle analysis

The assay was performed by Cell Cycle and Apoptosis Analysis Kit (Cat# BD0062-3, Bioworld, USA), according to the manufacturer’s instruction. Briefly, cells were fixed in 70% ethanol and then harvested to analyze cell cycle by flow cytometry after stained with PI/RNase staining buffer. The results were analyzed by Flowjo 7.6 software.

### Wound healing assay

Cells were seeded in six-well plates until confluent and then a 100 μl pipette tip was used for wound scraping on the cell monolayer and cultured in serum-free medium. Cells were photographed after 0 and 24 h to estimate the wound closure.

### Transwell invasion assay

In vitro transwell with Matrigel matrix (BD Biosciences, San Jose, CA) was applied to estimate the UBC cell invasion capacity. The mixture of Matrigel and PBS (1:8) was added to upper chambers and then incubated at 37 °C. After the gel solidified, 1.0 × 10^5^ cells in 100 μl serum-free medium were then seeded into the upper chambers allowed to invade, and the bottom well was added with the medium plus 10% FBS. After the incubation for 12 h (T24 cells), 16 h (UMUC-3 cells), or 20 h (5637 cells), transwells were fixed in 4% formaldehyde and stained with 0.2% crystal violet. The invaded cells were photographed and counted in three casually random fields.

### Sphere formation assay

A total of 1.0 × 10^5^ cells/well were seeded in the six-well ultra-low attachment plates (Corning, Steuben County, NY). After incubating in DMEM/F12 culture medium supplemented with 10 ng/ml human recombinant bFGF (PeproTech, Rocky Hill, NJ) and 10 ng/ml EGF (PeproTech) for 10–14 days. Sphere numbers were photographed and spheres with the diameter >50 μm were counted.

### Immunohistochemistry

A 5-μm-thick paraffin-embedded tumor sections from UBCs and nude mice were used for staining, followed by the procedures described previously^29^. Antibodies used were listed in Table S6. The expression levels were scored as intensity of the staining (0 = no staining; 1 = weak staining; 2 = moderate staining; 3 = intense staining) multiplied by percentage of the immunopositive staining area (1 = 0–25%; 2 = 25–49%; 3 = 50–75%; 4 = 75–100%). The median IHC score was chosen as the cutoff value for defining high and low expression.

### Bisulfite genomic sequencing

Genomic DNAs of human UBC tissues, adjacent non-tumorous tissues, and cell lines were isolated. The DNA was performed bisulfite conversion through The EpiMark Bisulfite Conversion Kit (Cat# E3318S, New England Biolabs). The bisulfite-converted DNA was immediately amplified with Champagne Taq DNA Polymerase (Cat# P122-d1, Vazyme, China). PCR products were cloned into pMD-19T (Cat #3271, TaKaRa, China) vector and processed to sequencing. The sequencing results were analyzed with BiQ Analyzer^44^. The gray level of each point represented the CpG island methylation level and was calculated by number of T vector clones with CG clones/(TG clones + CG clones). The primers of bisulfite sequencing amplification were described in Table S5.

### Transcriptome sequencing

Total RNAs in control or CBX7 overexpression T24 cells were extracted using Trizol reagent. Transcriptome sequencing was conducted using Illumina HiSeq instrument by MajorBio (Shanghai, China), and bioinformatics analysis was also performed. The RNA-seq data were uploaded with GEO accession no. GSE158244.

### Chromosome immunoprecipitation

ChIP assay was carried out by following the protocol of the ChIP assay kit (#17-371, Merck Millipore). Briefly, SDS lysis buffer was used to lyze 5637 cells and sonication was applied to shear DNA. Protein–DNA complexes were precipitated by CBX7, ubH2AK119, or control immunoglobulin G antibodies. The qRT-PCR was performed with primers specific for AKR1B10 promoter region. The primers used were listed in Table S5.

### In vivo xenograft models

Five-week-old male BALB/c nude mice were randomly categorized into control and experimental groups (*n* = 6/group). Stable UBC cells with CBX7 overexpression or depletion were subcutaneously injected into the dorsal flank of nude mice. Tumor size and body weight were measured every 4 days. Volumes were calculated using the following formula: volume = length × width^2^ × 0.5. All experimental procedures acquired official approval of the Institutional Animal Care and Use Committee of Model Animal Research Center of Nanjing University and the Institutional Animal Care and Use Committee of Shanghai Institute of Materia Medica, Chinese Academy of Sciences.

### Statistical analysis

All graphs were plotted and analyzed with GraphPad Prism 8.0 software. Values are expressed as mean ± SD. Between-groups comparisons were analyzed using Student’s *t* tests. A chi-square test was applied to evaluate the correlation between gene expression level and clinicopathological features. Pearson correlation was applied to measure the strength of association between DNA methylation and CBX7 expression. The OS was estimated by Kaplan–Meier (log-rank test) method. *p* > 0.05 was considered statistically nonsignificant (n.s.), and the following denotations were used: *p < 0.05; **p < 0.01, and ***p < 0.001.

## Supplementary information

Supplementary Figure legends

Supplementary Table 1

Supplementary Table 2

Supplementary Table 3

Supplementary Table 4

Supplementary Table 5

Supplementary Table 6

Supplementary Figure 1

Supplementary Figure 2

Supplementary Figure 3

Supplementary Figure 4

Supplementary Figure 5

Supplementary Figure 6

## References

[1] Siegel RL, Miller KD, Jemal A (2020). Cancer statistics, 2020. CA Cancer J. Clin..

[2] Chen W (2015). Cancer statistics: updated cancer burden in China. Chin. J. Cancer Res..

[3] Sternberg CN (2013). ICUD-EAU international consultation on bladder cancer 2012: chemotherapy for urothelial carcinoma-neoadjuvant and adjuvant settings. Eur. Urol..

[4] Yap KL, Zhou MM (2011). Structure and mechanisms of lysine methylation recognition by the chromodomain in gene transcription. Biochemistry.

[5] Chan HL, Morey L (2019). Emerging roles for polycomb-group proteins in stem cells and cancer. Trends Biochem. Sci..

[6] Zhou J (2014). Overexpression of HP1gamma is associated with poor prognosis in non-small cell lung cancer cell through promoting cell survival. Tumour Biol..

[7] Chang C (2018). A regulatory circuit HP1gamma/miR-451a/c-Myc promotes prostate cancer progression. Oncogene.

[8] Wotton D, Merrill JC (2007). Pc2 and SUMOylation. Biochem. Soc. Trans..

[9] Bracken AP, Helin K (2009). Polycomb group proteins: navigators of lineage pathways led astray in cancer. Nat. Rev. Cancer.

[10] Musselman CA, Lalonde ME, Cote J, Kutateladze TG (2012). Perceiving the epigenetic landscape through histone readers. Nat. Struct. Mol. Biol..

[11] Simon JA, Kingston RE (2009). Mechanisms of polycomb gene silencing: knowns and unknowns. Nat. Rev. Mol. Cell Biol..

[12] Shinjo K (2014). Expression of chromobox homolog 7 (CBX7) is associated with poor prognosis in ovarian clear cell adenocarcinoma via TRAIL-induced apoptotic pathway regulation. Int. J. Cancer.

[13] Zhang XW (2010). Oncogenic role of the chromobox protein CBX7 in gastric cancer. J. Exp. Clin. Cancer Res..

[14] Pallante P (2008). Loss of the CBX7 gene expression correlates with a highly malignant phenotype in thyroid cancer. Cancer Res..

[15] Karamitopoulou E (2010). Loss of the CBX7 protein expression correlates with a more aggressive phenotype in pancreatic cancer. Eur. J. Cancer.

[16] Kim HY, Park JH, Won HY, Lee JY, Kong G (2015). CBX7 inhibits breast tumorigenicity through DKK-1-mediated suppression of the Wnt/beta-catenin pathway. FASEB J..

[17] Pallante P (2010). The loss of the CBX7 gene expression represents an adverse prognostic marker for survival of colon carcinoma patients. Eur. J. Cancer.

[18] Baylin SB, Jones PA (2011). A decade of exploring the cancer epigenome - biological and translational implications. Nat. Rev. Cancer.

[19] Torii S, Yamamoto T, Tsuchiya Y, Nishida E (2006). ERK MAP kinase in G cell cycle progression and cancer. Cancer Sci..

[20] Neuzillet C (2014). MEK in cancer and cancer therapy. Pharm. Ther..

[21] Wei Y (2016). Benzo[a]pyrene promotes gastric cancer cell proliferation and metastasis likely through the Aryl hydrocarbon receptor and ERK-dependent induction of MMP9 and c-myc. Int. J. Oncol..

[22] Judd NP (2012). ERK1/2 regulation of CD44 modulates oral cancer aggressiveness. Cancer Res..

[23] Siu MKY (2019). Hexokinase 2 regulates ovarian cancer cell migration, invasion and stemness via FAK/ERK1/2/MMP9/NANOG/SOX9 signaling cascades. Cancers.

[24] Takemura M (2011). Selective inhibition of the tumor marker aldo-keto reductase family member 1B10 by oleanolic acid. J. Nat. Prod..

[25] Zhang W, Li H, Yang Y, Liao J, Yang GY (2014). Knockdown or inhibition of aldo-keto reductase 1B10 inhibits pancreatic carcinoma growth via modulating Kras-E-cadherin pathway. Cancer Lett..

[26] Huo W, Tan D, Chen Q (2020). CASC9 facilitates cell proliferation in bladder cancer by regulating CBX2 expression. Nephron.

[27] Yuan GJ (2017). Chromobox homolog 8 is a predictor of muscle invasive bladder cancer and promotes cell proliferation by repressing the p53 pathway. Cancer Sci..

[28] Nawaz Z (2016). Cbx7 is epigenetically silenced in glioblastoma and inhibits cell migration by targeting YAP/TAZ-dependent transcription. Sci. Rep..

[29] Yan YL (2020). Downregulation of TET1 promotes bladder cancer cell proliferation and invasion by reducing DNA hydroxymethylation of AJAP1. Front. Oncol..

[30] Nunes SP, Henrique R, Jeronimo C, Paramio JM (2020). DNA methylation as a therapeutic target for bladder cancer. Cells.

[31] Zeng JS (2018). CBX4 exhibits oncogenic activities in breast cancer via Notch1 signaling. Int. J. Biochem. Cell Biol..

[32] Dietrich N (2007). Bypass of senescence by the polycomb group protein CBX8 through direct binding to the INK4A-ARF locus. EMBO J..

[33] Federico A (2009). Chromobox protein homologue 7 protein, with decreased expression in human carcinomas, positively regulates E-cadherin expression by interacting with the histone deacetylase 2 protein. Cancer Res..

[34] Federico A (2019). The complex CBX7-PRMT1 has a critical role in regulating E-cadherin gene expression and cell migration. Biochim. Biophys. Acta Gene Regul. Mech..

[35] Shi J (2019). Aldo-keto reductase family 1 member B10 (AKR1B10) overexpression in tumors predicts worse overall survival in hepatocellular carcinoma. J. Cancer.

[36] Ma J (2012). AKR1B10 overexpression in breast cancer: association with tumor size lymph node metastasis and patient survival and its potential as a novel serum marker. Int. J. Cancer.

[37] Chung YT (2012). Overexpression and oncogenic function of aldo-keto reductase family 1B10 (AKR1B10) in pancreatic carcinoma. Mod. Pathol..

[38] Fukumoto S (2005). Overexpression of the aldo-keto reductase ffamily protein AKR1B10 is highly correlated with smokers’ nonsmall cell lung carcinomas. Clin. Cancer Res..

[39] Hashimoto Y (2013). Carboplatin-gemcitabine combination chemotherapy upregulates AKR1B10 expression in bladder cancer. Int. J. Clin. Oncol..

[40] Li J (2017). AKR1B10 promotes breast cancer cell migration and invasion via activation of ERK signaling. Oncotarget.

[41] Cong Z (2019). Long non-coding RNA linc00665 promotes lung adenocarcinoma progression and functions as ceRNA to regulate AKR1B10-ERK signaling by sponging miR-98. Cell Death Dis..

[42] Robertson AG (2017). Comprehensive molecular characterization of muscle-invasive bladder cancer. Cell.

[43] Dalangood S (2020). Identification of glycogene-type and validation of ST3GAL6 as a biomarker predicts clinical outcome and cancer cell invasion in urinary bladder cancer. Theranostics.

[44] Bock C (2005). BiQ Analyzer: visualization and quality control for DNA methylation data from bisulfite sequencing. Bioinformatics.

