# Supplementary material for: CBX7 suppresses urinary bladder cancer progression via modulating AKR1B10–ERK signaling
Source: Cell Death Dis. 2021 May 25;12(6):537. doi: 10.1038/s41419-021-03819-0 (PMC8149849; doi:10.1038/s41419-021-03819-0)
Supplement: Supplementary file 1 — Supplementary Figure legends [file 41419_2021_3819_MOESM1_ESM.docx]

**Supplementary Figure 1. a.** CBX7 mRNA expression between UBC tissues and the adjacent normal tissues in GSE19915. **b, c.** The association of CBX7 mRNA expression with clinicopathologic features in GSE19915. **d.** Kaplan-Meier plot of overall survival of UBC patients in GSE19915. **e.** The representative images for different IHC staining intensities of CBX7 in UBC patients. Scale bar, 100 μm (upper panel) and 20 μm (lower panel).

**Supplementary Figure 2. a.** Ectopic expression of CBX7 and cell cycle related regulators was detected in UMUC-3 cells by Western blotting. **b, c.** Effects of CBX7 overexpression on cell proliferation and colony formation. **d, e.** Effects of CBX7 overexpression on cell cycle were determined by flow cytometry analysis. Data are presented as means ± SD from three independent replicates. *p<0.05, ***p<0.001, ns, non-significant.

**Supplementary Figure 3. a-c.** Effects of CBX7 overexpression on cell invasion (**a**) and migration (**b**, **c**). Scale bar, 100 μm (**a**) and 200 μm (**b**, **e**). **d.** Epithelial and mesenchymal transition markers, and matrix metalloproteinases were detected by Western blotting. **e.** Effects of CBX7 overexpression on the size and sphere number. Scale bar, 100 μm. **f.** The levels of core stem cell genes were detected by Western blotting. Data are presented as means ± SD from three independent replicates. ***p<0.001.

**Supplementary Figure 4. a-c.** The altered mRNA levels of genes were selectively confirmed by qRT-PCR upon CBX7 overexpression or knockdown. **d.** The association of AKR1B10 mRNA expression with grade in GSE13507. **e.** The representative images for different IHC staining intensities of AKRAB0 in UBC patients. Scale bar, 100 μm (upper panel) and 20 μm (lower panel).

**Supplementary Figure 5.** **a-d.** H&E staining was used to examine UBC cell morphologies, IHC staining for CBX7 examined CBX7 expression and Ki-67 staining revealed cell proliferation in UMUC-3 and 5637 xenografts by counting the proportion of Ki-67 positive cells. Scale bar, 20 μm. Data are presented as means ± SD from six independent replicates

**Supplementary Figure 6.** The whole untrimmed Western blotting images were presented.

**Supplementary Table 1.** The association between CBX7 protein levels and clinicpatholo -gical features of UBC patients

**Supplementary Table 2.** Univariate and multivariate analysis of clinicopathological features and survival time of UBC patients

**Supplementary Table 3.** The association between AKR1B10 protein levels and clinicopathological features of UBC patients

**Supplementary Table 4.** List of chemicals and kits.

**Supplementary Table 5.** List for sequences of primer sets, shRNAs and siRNAs.

**Supplementary Table 6.** List of antibodies.
