# Supplementary material for: CBX7 suppresses urinary bladder cancer progression via modulating AKR1B10–ERK signaling
Source: Cell Death Dis. 2021 May 25;12(6):537. doi: 10.1038/s41419-021-03819-0 (PMC8149849; doi:10.1038/s41419-021-03819-0)
Supplement: Supplementary file 2 — Supplementary Table 1 [file 41419_2021_3819_MOESM2_ESM.docx]

**Table 1** **The association between CBX7 protein levels and clinicopathological features of UBC patients (n=81)**

| **Characteristics** | **Number** | **Expression of CBX7** | | **p value** |
| --- | --- | --- | --- | --- |
|  |  | **high（n，%）** | **low（n，%）** |  |
| Gender |  |  |  | 0.464 |
| male | 65 | 34(52.3%) | 31(47.7%) |  |
| female | 16 | 10(62.5%) | 6(37.5%) |  |
| Age |  |  |  | 0.253 |
| ≥60 | 47 | 23(48.9%) | 24(51.1%) |  |
| <60 | 34 | 21(61.8%) | 13(38.2%) |  |
| T stage |  |  |  | **＜0.001** |
| Ta-1 | 44 | 35(79.5%) | 9(20.5%) |  |
| T2-4 | 37 | 9(24.3%) | 28(75.7%) |  |
| Tumor grade |  |  |  | **0.002** |
| Low | 25 | 20(80.0%) | 5(20.0%) |  |
| High | 56 | 24(36.9%) | 32(57.1%) |  |

Numbers in bold indicate p value with statistical difference.
