# Supplementary material for: CBX7 suppresses urinary bladder cancer progression via modulating AKR1B10–ERK signaling
Source: Cell Death Dis. 2021 May 25;12(6):537. doi: 10.1038/s41419-021-03819-0 (PMC8149849; doi:10.1038/s41419-021-03819-0)
Supplement: Supplementary file 3 — Supplementary Table 2 [file 41419_2021_3819_MOESM3_ESM.docx]

**Table 2 Univariate and multivariate analysis of clinicopathological features and survival time of UBC patients (n=81)**

| **Variables** | **Univariate analysis** | |  | | **Multivariate analysis** | |
| --- | --- | --- | --- | --- | --- | --- |
|  | **HR (95% CI)** | **p value** | | **HR (95% CI)** | | **p value** |
| Age (year)  (<60 vs. ≥60) | 1.572  (0.787 – 3.139) | 0.120 | | 2.025  (0.975 – 4.208) | | 0.059 |
| Gender  (female vs. male) | 1.330  (0.583 – 3.034) | 0.499 | | 1.090  (0.468 – 2.536) | | 0.842 |
| T stage  (Ta-1 vs. T2-4) | 1.866  1.381 – 2.522 | **<0.001** | | 1.462  (1.015 – 2.107) | | **0.041** |
| Grade  (low vs. high) | 4.901  1.893 – 12.694 | **0.001** | | 3.435  (1.277 – 9.241) | | **0.015** |
| CBX7 expression  (low vs. high) | 0.835  0.761 – 0.917 | **<0.001** | | 0.869  (0.780 – 0.967) | | **0.011** |

HR, hazard ratio; CI, confidence interval. Numbers in bold indicate p value with statistical difference.
