# Supplementary material for: CBX7 suppresses urinary bladder cancer progression via modulating AKR1B10–ERK signaling
Source: Cell Death Dis. 2021 May 25;12(6):537. doi: 10.1038/s41419-021-03819-0 (PMC8149849; doi:10.1038/s41419-021-03819-0)
Supplement: Supplementary file 4 — Supplementary Table 3 [file 41419_2021_3819_MOESM4_ESM.docx]

**Table 3 The association between AKR1B10 protein levels and clinicopathological features of UBC patients (n=70)**

| **Characteristics** | **Number** | **Expression of AKR1B10** | | **p value** |
| --- | --- | --- | --- | --- |
|  |  | **High（n，%）** | **Low（n，%）** |  |
| Gender |  |  |  | 0.718 |
| male | 61 | 22(36.1%) | 39(63.9%) |  |
| female | 9 | 4(44.4%) | 5(55.6%) |  |
| Age |  |  |  | >0.999 |
| ≥60 | 46 | 17(37.0%) | 29(63.0%) |  |
| <60 | 24 | 9(37.5%) | 15(62.5%) |  |
| Tumor grade |  |  |  | **0.005** |
| Low | 26 | 4(15.4%) | 22(84.6%) |  |
| High | 44 | 22(50.0%) | 22(50.0%) |  |
| T stage |  |  |  | 0.326 |
| Ta-1 | 30 | 9(30.0%) | 21(70.0%) |  |
| T2-4 | 40 | 17(42.5%) | 23(57.5%) |  |
| N stage |  |  |  | 0.551 |
| N0 | 67 | 24(35.8%) | 43(64.2%) |  |
| ≥N1 | 3 | 2(66.7%) | 1(33.3%) |  |
| M stage |  |  |  | 0.108 |
| M0 | 49 | 15(30.6%) | 34(69.4%) |  |
| ≥M1 | 21 | 11(52.4%) | 10(47.6%) |  |

Numbers in bold indicate p value with statistical difference.
