# Supplementary material for: CBX7 suppresses urinary bladder cancer progression via modulating AKR1B10–ERK signaling
Source: Cell Death Dis. 2021 May 25;12(6):537. doi: 10.1038/s41419-021-03819-0 (PMC8149849; doi:10.1038/s41419-021-03819-0)
Supplement: Supplementary file 5 — Supplementary Table 4 [file 41419_2021_3819_MOESM5_ESM.docx]

**Table S1 List of chemicals and kits**

| **Items** | **Company** | **Cat. No.** |
| --- | --- | --- |
| **Chemicals** |  |  |
| 5-Aza-CdR | Sigma-Aldrich | A3656 |
| bFGF | PeproTech | 100-18B-500 |
| Crystal violet | Sangon Biotech | A100528 |
| EGF | PeproTech | AF-100-15 |
| MTT | Beyotime | ST316 |
| Matrigel gel | BD Biosciences | 356230 |
| Oleanolic acid | MedChemExpress | HY-N0156 |
| PRT4165 | MedChemExpress | HY-19817 |
| Puromycin | Yeason | ISY1130 |
| **Reagents and Kits** |  |  |
| Cell Cycle and Apoptosis Analysis Kit | Bioworld | BD0062-3 |
| Champagne Taq DNA Polymerase | Vazyme | P122-d1 |
| ChIP assay kit | Merck Millipore | #17-371 |
| EpiMark Bisulfite Conversion Kit | New England Biolabs | E3318S |
| pMD-19T | TaKaRa | 3271 |
| Prime-Script RT-PCR kit | TaKaRa | RR047A |
| TRIzol reagent | TaKaRa | 9109 |
