# Supplementary material for: CBX7 suppresses urinary bladder cancer progression via modulating AKR1B10–ERK signaling
Source: Cell Death Dis. 2021 May 25;12(6):537. doi: 10.1038/s41419-021-03819-0 (PMC8149849; doi:10.1038/s41419-021-03819-0)
Supplement: Supplementary file 6 — Supplementary Table 5 [file 41419_2021_3819_MOESM6_ESM.docx]

**Table S2 List for sequences of primer sets, shRNAs and siRNAs**

|  | **Direction** | **Sequences (5’-3’)** |
| --- | --- | --- |
| **Primer sets for qRT-PCR** |  |  |
| *β-actin* | Forward | CATGTACGTTGCTATCCAGGC |
|  | Reverse | CTCCTTAATGTCACGCACGAT |
| *AKR1B10* | Forward | AACGTGTTGCAATCCTCTCA |
|  | Reverse | TGGGACATGAGTGGAGGTAGT |
| *CBX7* | Forward | GCGTGCGGAAGGGTAAAGT |
|  | Reverse | GCTTGGGTTTCGGACCTCTC |
| *DNMT1* | Forward | CCTAGCCCCAGGATTACAAGG |
|  | Reverse | ACTCATCCGATTTGGCTCTTTC |
| *DNMT3A* | Forward | AGTACGACGACGACGGCTA |
|  | Reverse | CACACTCCACGCAAAAGCAC |
| *DNMT3B* | Forward | ACCTCGTGTGGGGAAAGATCA |
|  | Reverse | CCATCGCCAAACCACTGGA |
| *Ring 1b* | Forward | CAAACGGAACTCAACCATTAAGC |
|  | Reverse | CCACTTCTAAGGGCTGTGATG |
| **Primer sets for Bisulfite DNA sequencing** | | |
| CBX7 promoter-449F |  | AAAGGTTAAAGTTTAGTAGGAAAAAT |
| CBX7 promoter-449R |  | CTCCCAAAACCCCAATAAAAT |
| **Primer sets for ChIP analysis** | | |
| AKR1B10 promoter-P1F |  | GTTCTGAGACCGGCTTAT |
| AKR1B10 promoter-P1R |  | TTCCTTCCAGAACCACTTAG |
| AKR1B10 promoter-P2F |  | CATGCATATTCCCATTGCAG |
| AKR1B10 promoter-P2R |  | CTTGCGGCACTTTATTTTCC |
| AKR1B10 promoter-P3F |  | TCCCAAAGTGCTGGGATTAC |
| AKR1B10 promoter-P3R |  | TGTTGATGAGTTGGCTTTGG |
| AKR1B10 promoter-P4F |  | TTCTCAGCCTCCCAAGTAGC |
| AKR1B10 promoter-P4R |  | AAGGTAGGGAGGGAGACAGC |
| **PCR for shRNA subcloning** |  |  |
| shCBX7-1F |  | CCGGGCCAGAAGAGCACATCTTGTTCAAGAGA  CAAGATGTGCTCTTCTGGCTTTTTG |
| shCBX7-1R |  | AATTCAAAAAAGCCAGAAGAGCACATCTTG  TCTCTTGAACAAGATGTGCTCTTCTGGC |
| shCBX7-2F |  | CCGGTCATGGCCTACGAGGAGAATTCAAGAGA  TTCTCCTCGTAGGCCATGATTTTTG |
| shCBX7-2R |  | AATTCAAAAAATCATGGCCTACGAGGAGAA  TCTCTTGAATTCTCCTCGTAGGCCATGA |
| **siRNA sequences** |  |  |
| siNC |  | UUCUCCGAACGUGUCACGU |
| siAKR1B10 |  | GCCUGUAACGUGUUGCAAUTT |
| siDNMT1 |  | GCACAGAAGUCAACCCAAAUU |
| siDNMT3A |  | CCUCAAGAGCAGUGGAAAATT |
| **(Cont’d)** |  |  |
|  | **Direction** | **Sequences (5’-3’)** |
| **siRNA sequences** |  |  |
| siDNMT3B |  | GCAUAAAGGUAGGAAAGUAUU |
| siRing 1b |  | CUAGAGCUUGAUAAUAACATT |

NC, negative control.
