# Supplementary material for: CBX7 suppresses urinary bladder cancer progression via modulating AKR1B10–ERK signaling
Source: Cell Death Dis. 2021 May 25;12(6):537. doi: 10.1038/s41419-021-03819-0 (PMC8149849; doi:10.1038/s41419-021-03819-0)
Supplement: Supplementary file 7 — Supplementary Table 6 [file 41419_2021_3819_MOESM7_ESM.docx]

**Table S3 List of antibodies**

| **Antibody** | **Company** | **Cat. No.** | **Dilution (Application)** |
| --- | --- | --- | --- |
| ACTIN | Abclonal | AC206 | 1:2,000 (WB) |
| Anti-Flag | Abclonal | AE-005 | 1:1,000 (WB) |
| Anti-HA | Santa Cruz | sc-7392 | 1:1,000 (WB) |
| Active β-catenin | Cell Signaling Technology | 8814S | 1:1,000 (WB) |
| β-catenin | Cell Signaling Technology | 9581S | 1:1,000 (WB) |
| ALDH1A3 | Proteintech | 25167-1-AP | 1:1,000 (WB) |
| AKR1B10 | Proteintech | 18252-1-AP | 1:1,000 (WB) |
| AKR1B10 | Abcam | ab192865 | 1:1,000 (IHC) |
| AKT | Cell Signaling Technology | 9272S | 1:1,000 (WB) |
| p-AKT | Cell Signaling Technology | 4060S | 1:1,000 (WB) |
| CBX7 | Abcam | ab21873 | 1:1,000 (WB, IHC) |
| CD44 | Cell Signaling Technology | 156-3C11 | 1:1,000 (WB) |
| CDK6 | Proteintech | 14052-1-AP | 1:1,000 (WB) |
| Cyclin D1 | Santa Cruz | sc753 | 1:1,000 (WB) |
| DNMT1 | Santa Cruz | sc-20701 | 1:1,000 (WB) |
| DNMT3A | Santa Cruz | sc-20703 | 1:1,000 (WB) |
| E-Cadherin | Proteintech | 20874-1-AP | 1:1,000 (WB) |
| ERK | Cell Signaling Technology | 4695 | 1:2,000 (WB) |
| p-ERK | Cell Signaling Technology | 4370P | 1:1,000 (WB) |
| H2A | Cell Signaling Technology | 12349S | 1:1,000 (WB) |
| ubH2AK119 | Cell Signaling Technology | 8240S | 1:1,000 (WB) |
| Ki67 | Abcam | ab15580 | 1:1,000 (IHC) |
| MMP-2 | Cell Signaling Technology | 40994S | 1:1,000 (WB) |
| MMP-9 | Abclonal | A2095 | 1:1,000 (WB) |
| NANOG | Proteintech | 14295-1-AP | 1:1,000 (WB) |
| N-Cadherin | Proteintech | 22018-1-AP | 1:1,000 (WB) |
| PCNA | Santa Cruz | sc-56 | 1:1,000 (WB) |
| p21 | Santa Cruz | sc-397 | 1:1,000 (WB) |
| SOX2 | Cell Signaling Technology | D988N | 1:1,000 (WB) |
| STAT3 | Cell Signaling Technology | 9139S | 1:1,000 (WB) |
| p-STAT3 | Cell Signaling Technology | 9145S | 1:1,000 (WB) |
| Vimentin | Proteintech | 10336-1-AP | 1:2000 (WB) |
| 2nd antibody mouse IgG | Cell Signaling Technology | 7076s | 1:5,000 (WB) |
| 2nd antibody rabbit IgG | Cell Signaling Technology | 7074s | 1:5,000 (WB) |
