# Supplementary figures and images for: CBX7 suppresses urinary bladder cancer progression via modulating AKR1B10–ERK signaling
Source: Cell Death Dis. 2021 May 25;12(6):537. doi: 10.1038/s41419-021-03819-0 (PMC8149849; doi:10.1038/s41419-021-03819-0)

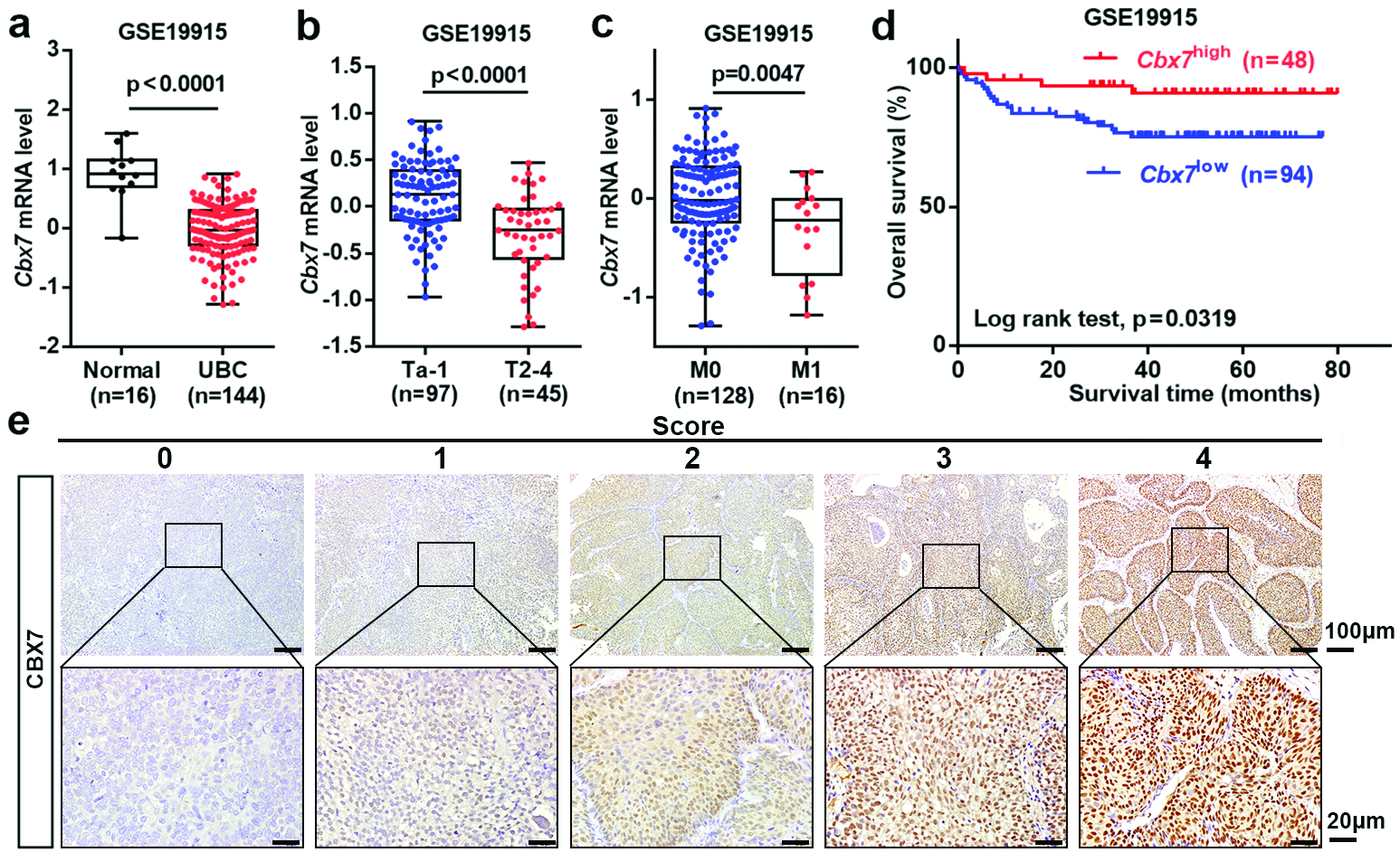

Supplement: Supplementary file 8 — Supplementary Figure 1 [file 41419_2021_3819_MOESM8_ESM.tif]

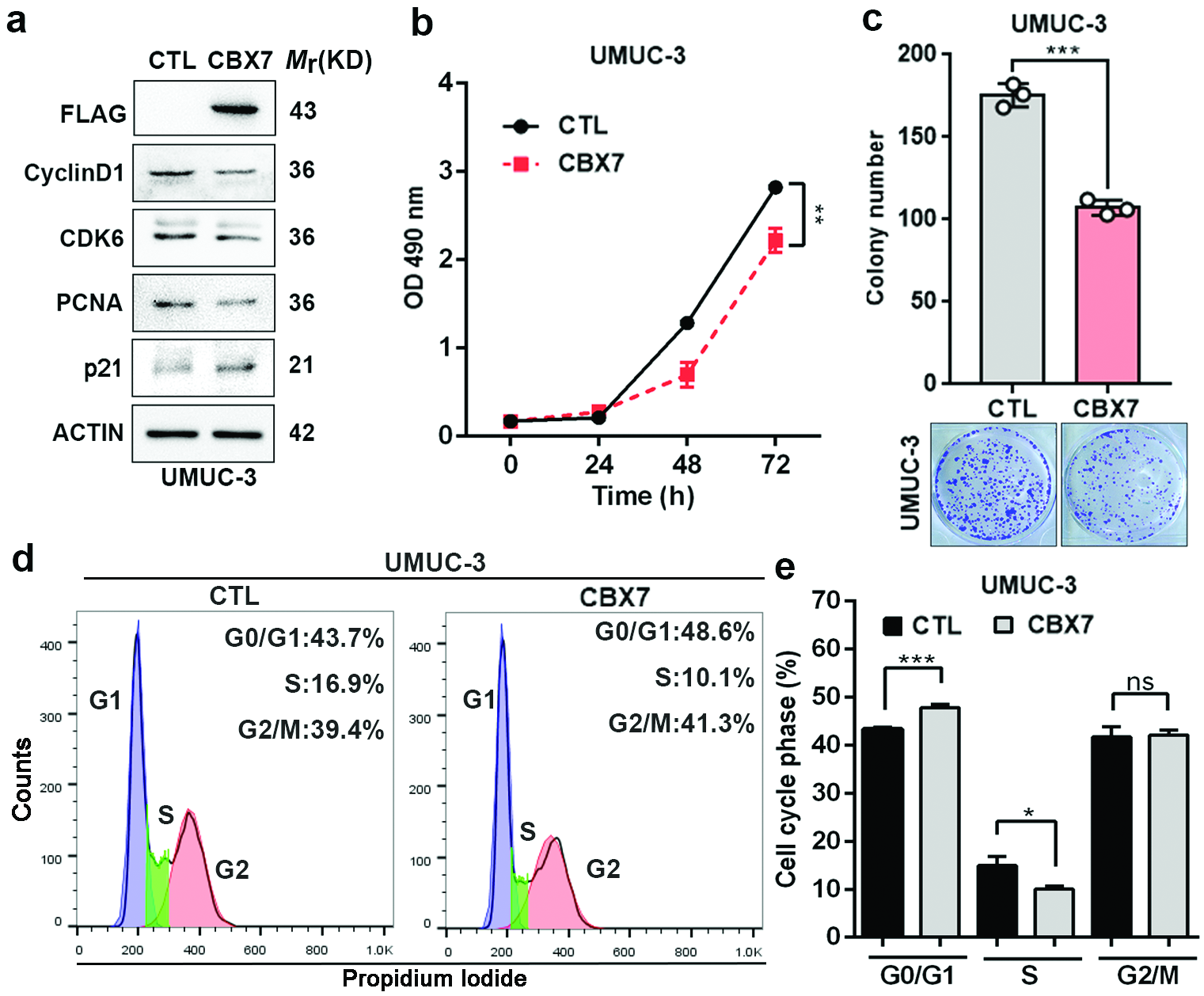

Supplement: Supplementary file 9 — Supplementary Figure 2 [file 41419_2021_3819_MOESM9_ESM.tif]

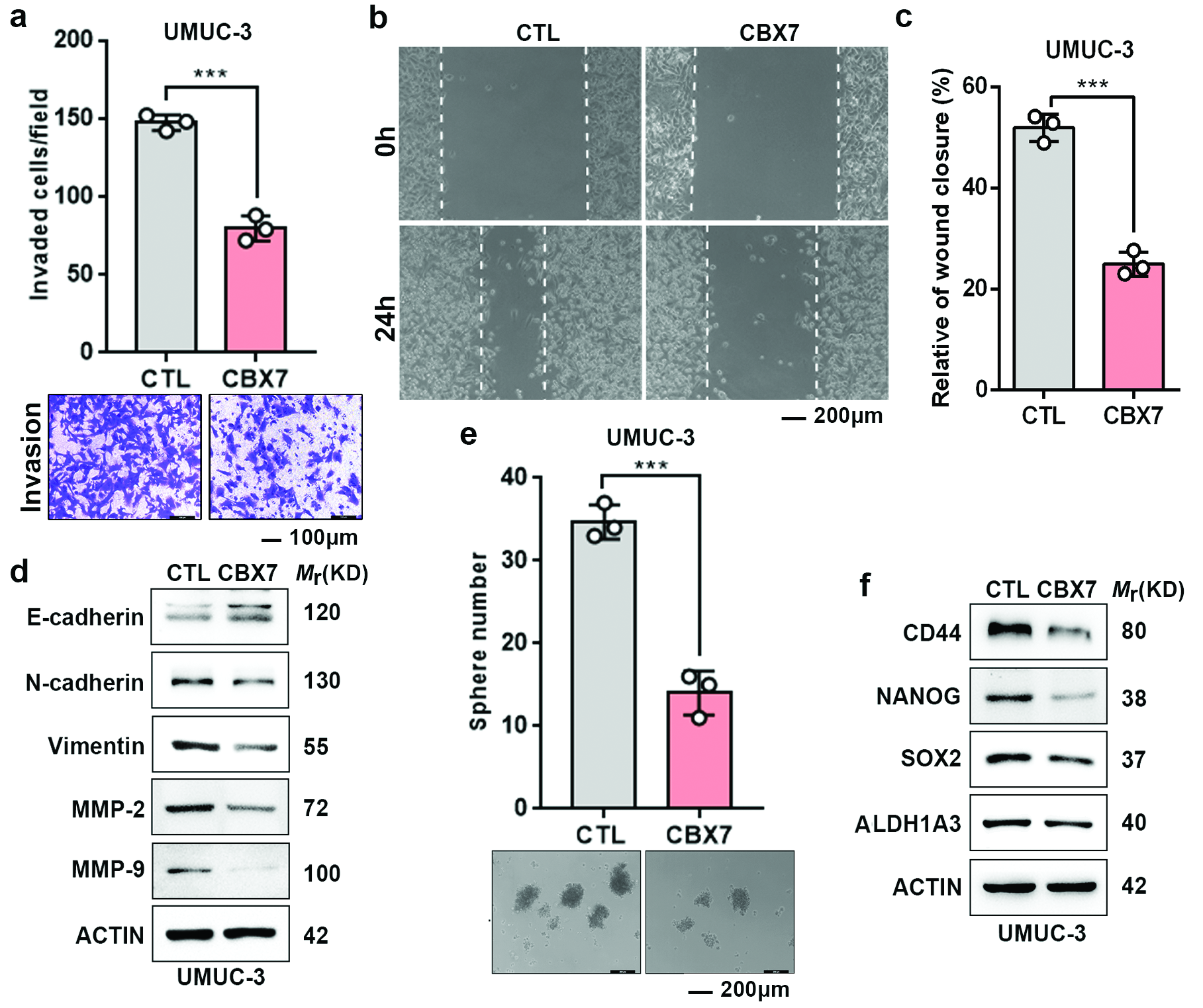

Supplement: Supplementary file 10 — Supplementary Figure 3 [file 41419_2021_3819_MOESM10_ESM.tif]

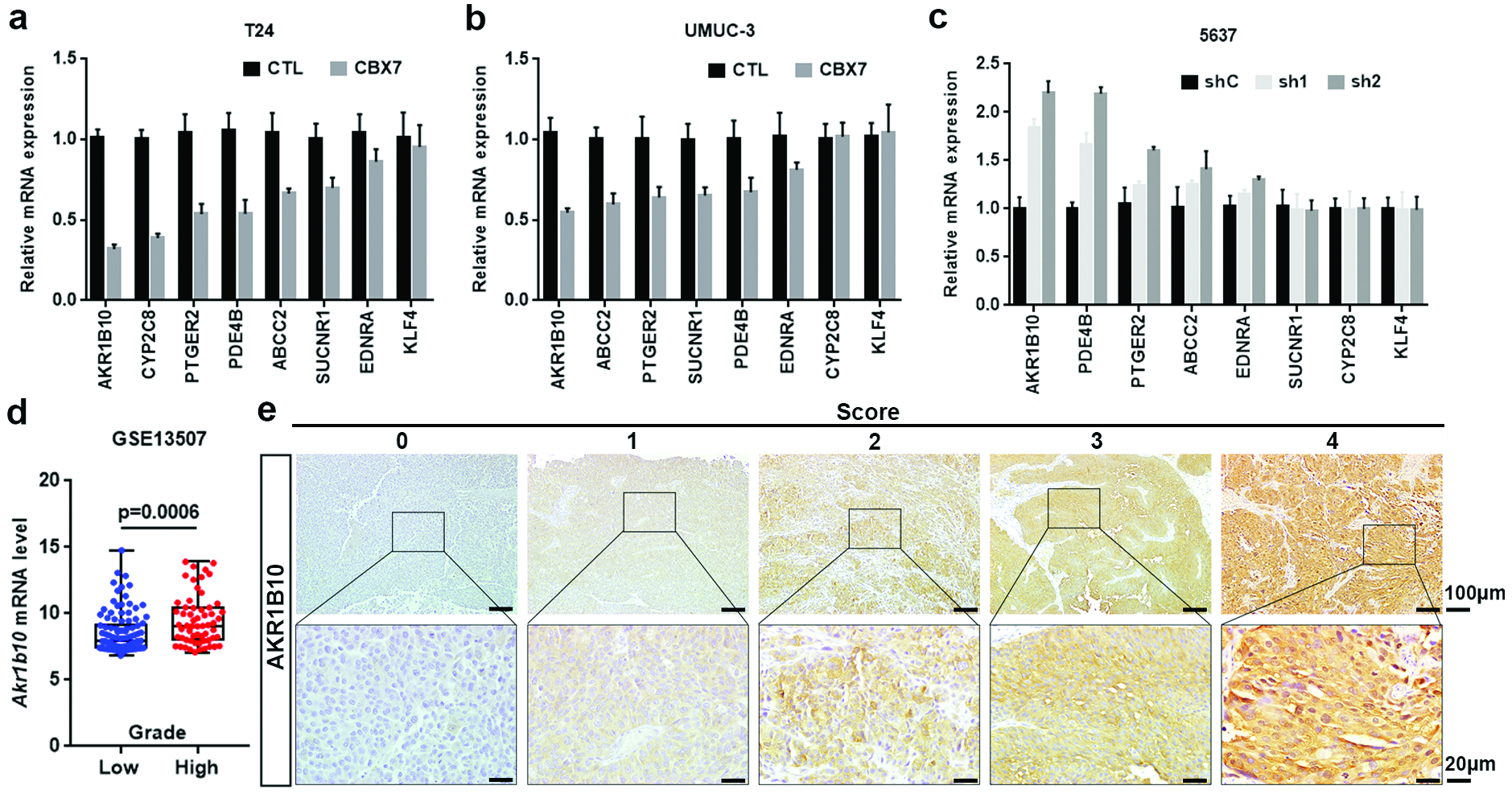

Supplement: Supplementary file 11 — Supplementary Figure 4 [file 41419_2021_3819_MOESM11_ESM.tif]

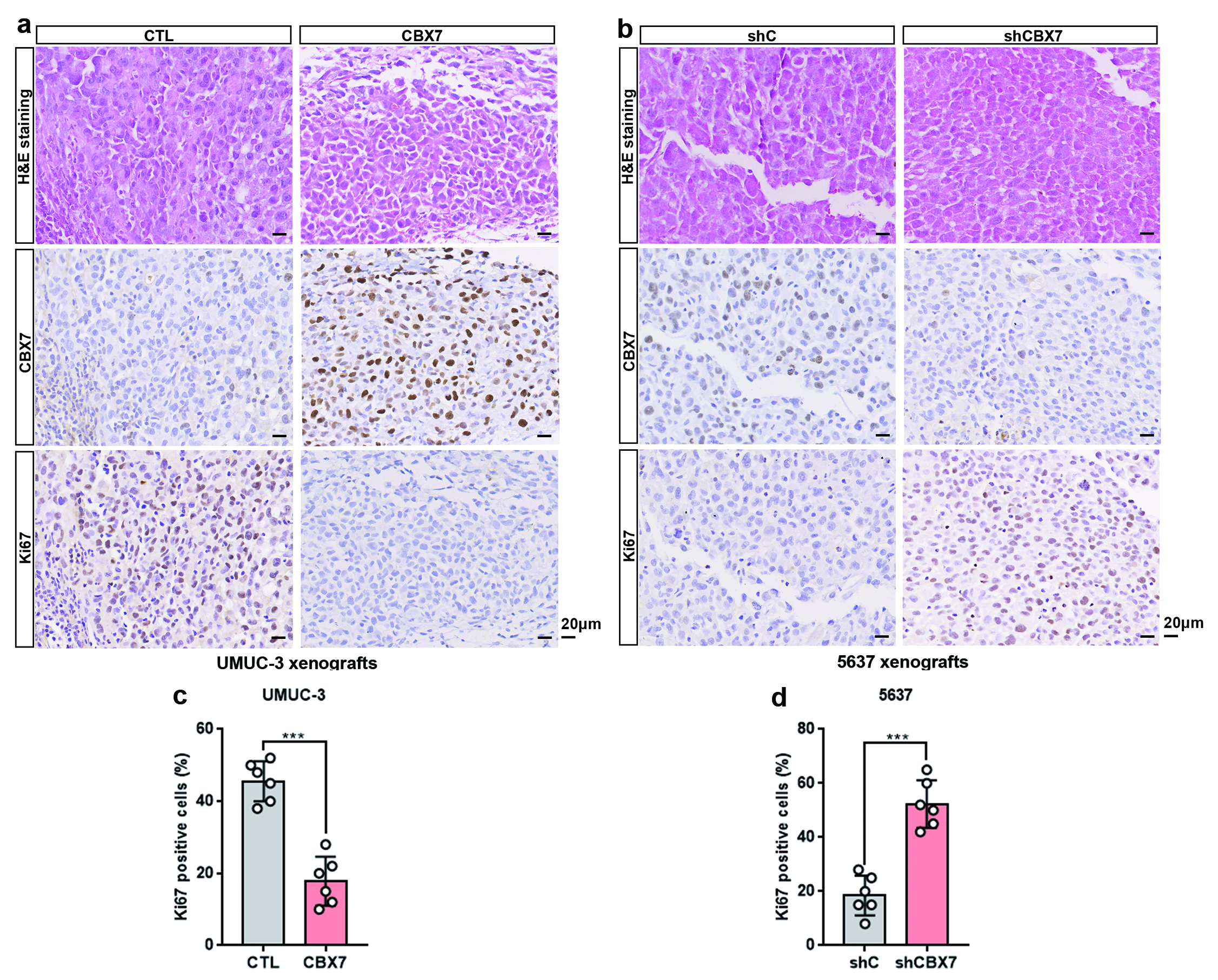

Supplement: Supplementary file 12 — Supplementary Figure 5 [file 41419_2021_3819_MOESM12_ESM.tif]

**Fig.1k**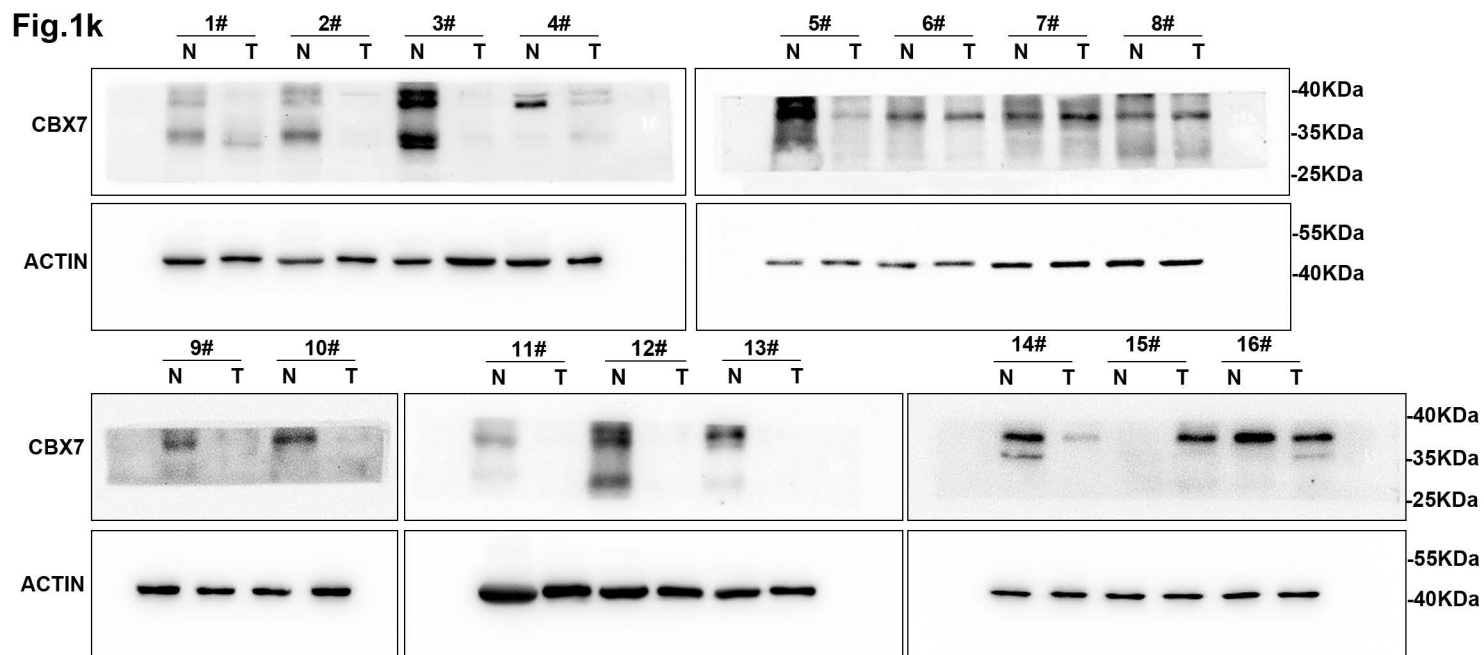**Fig.1o**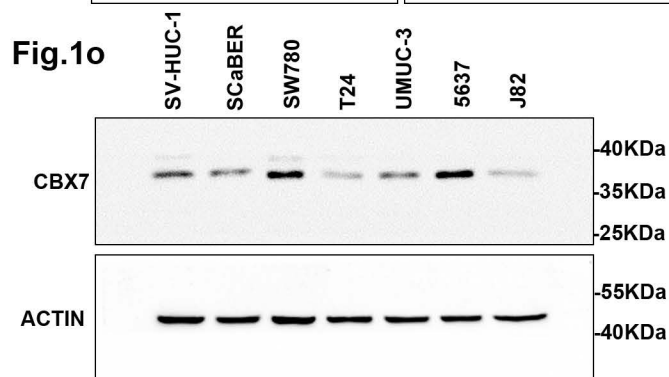

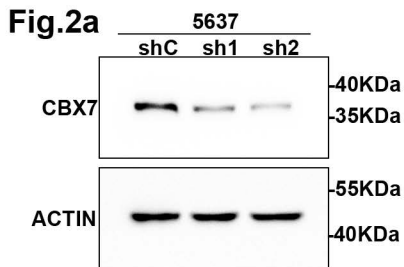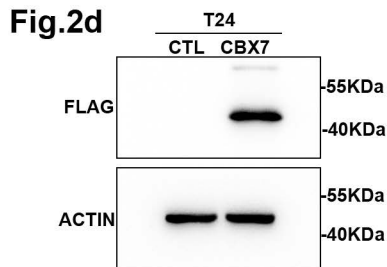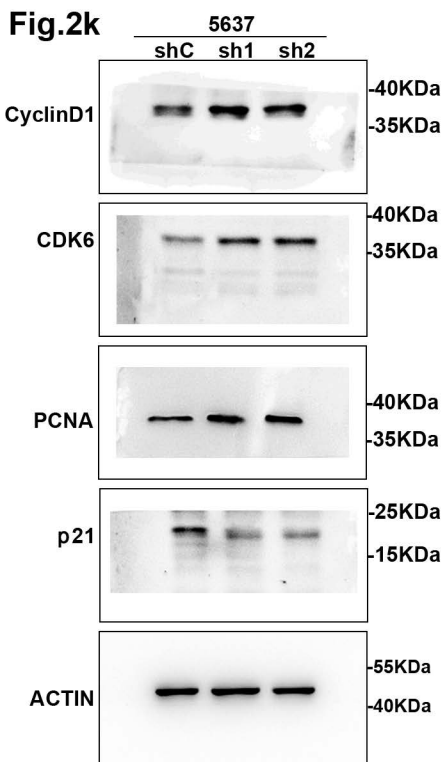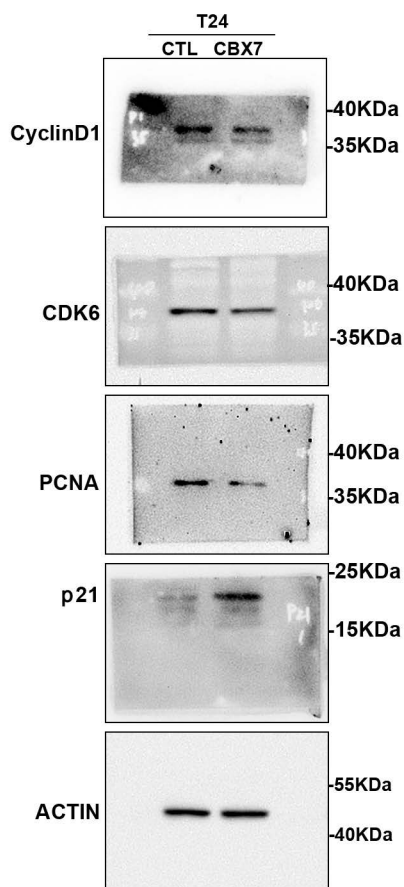

**Fig.3g**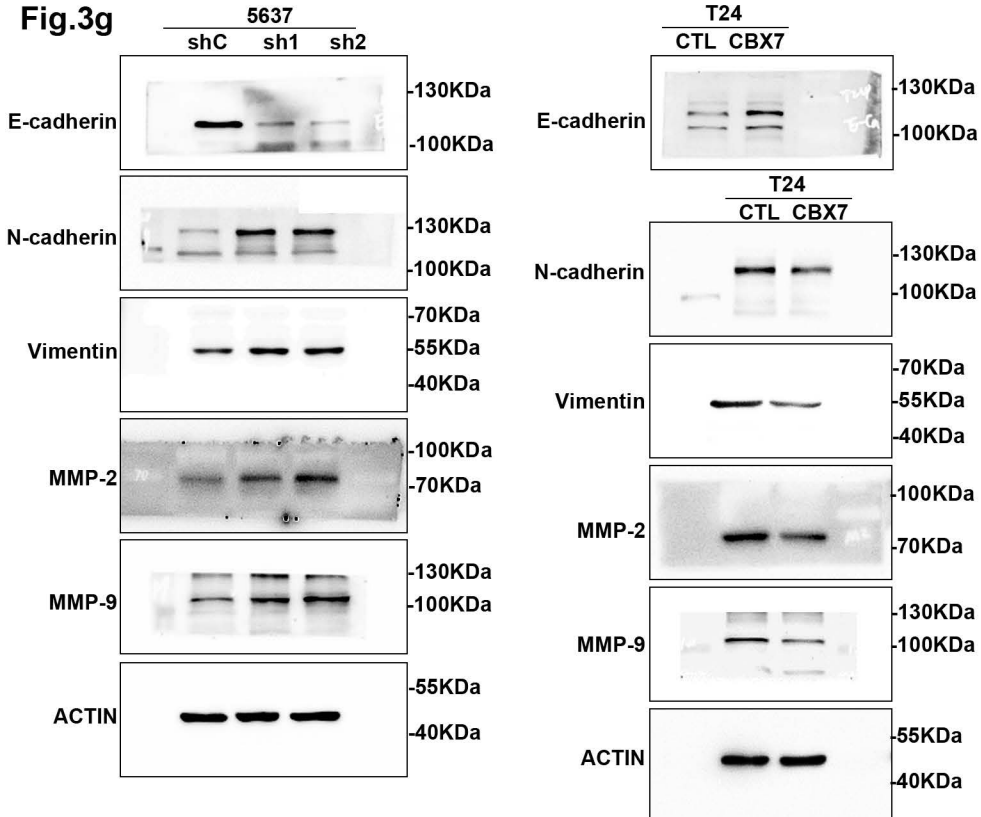**Fig.3j**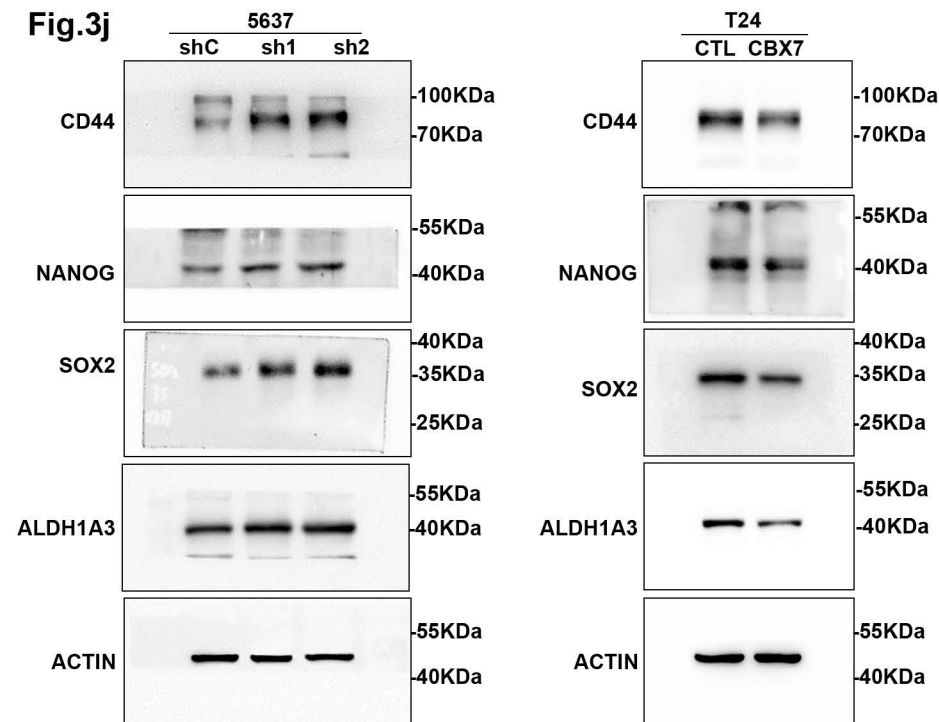

**Fig.4d**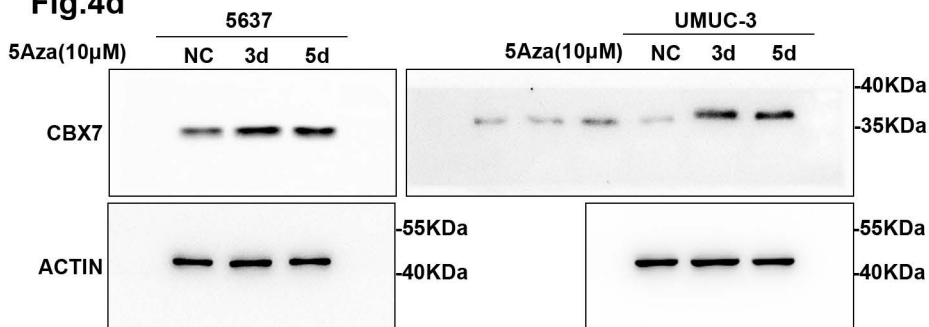**Fig.4h**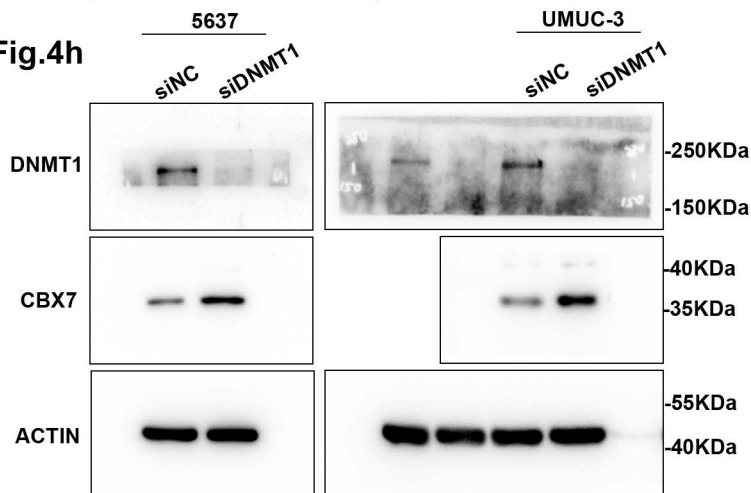**Fig.4j**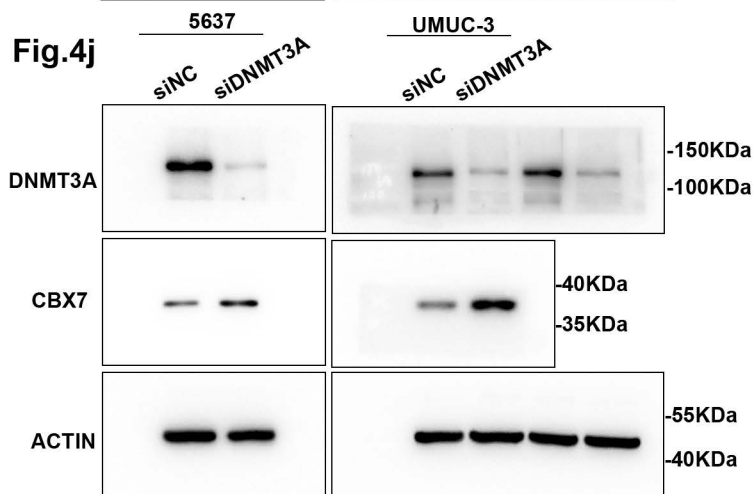

**Fig.5c**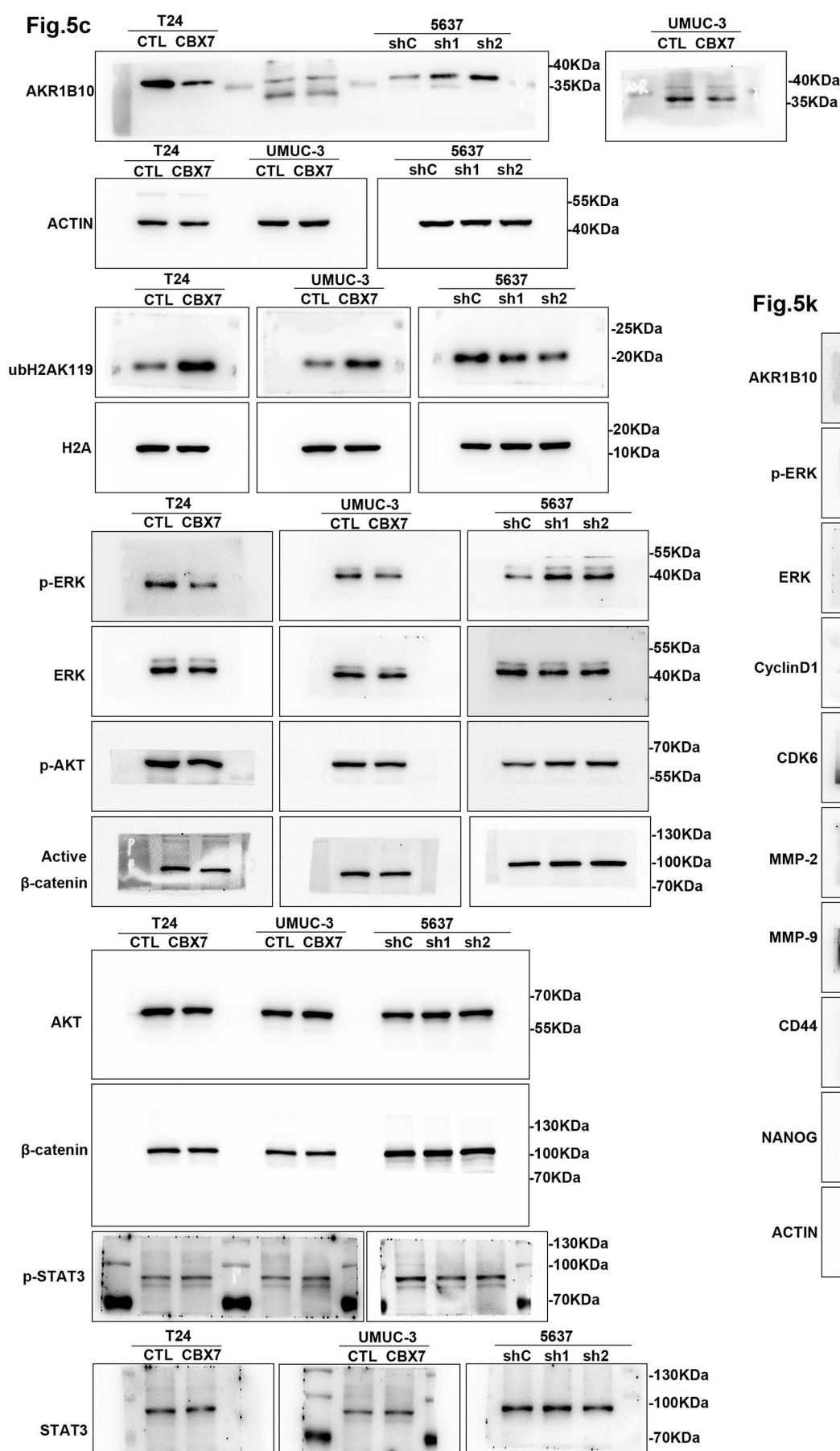**Fig.5k**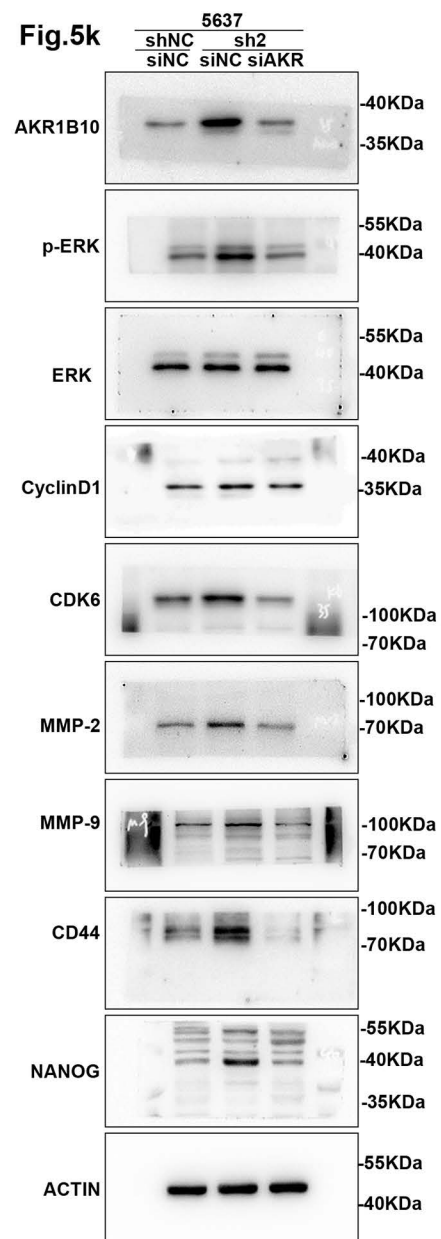

**Fig.6a**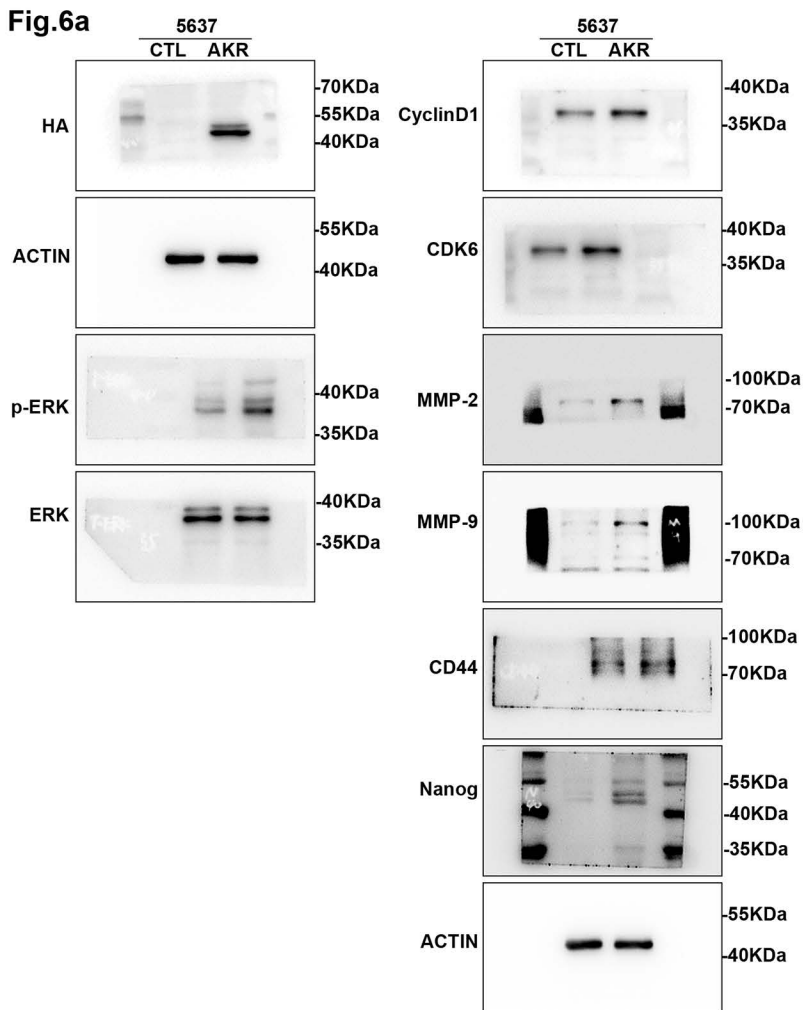**Fig.6g**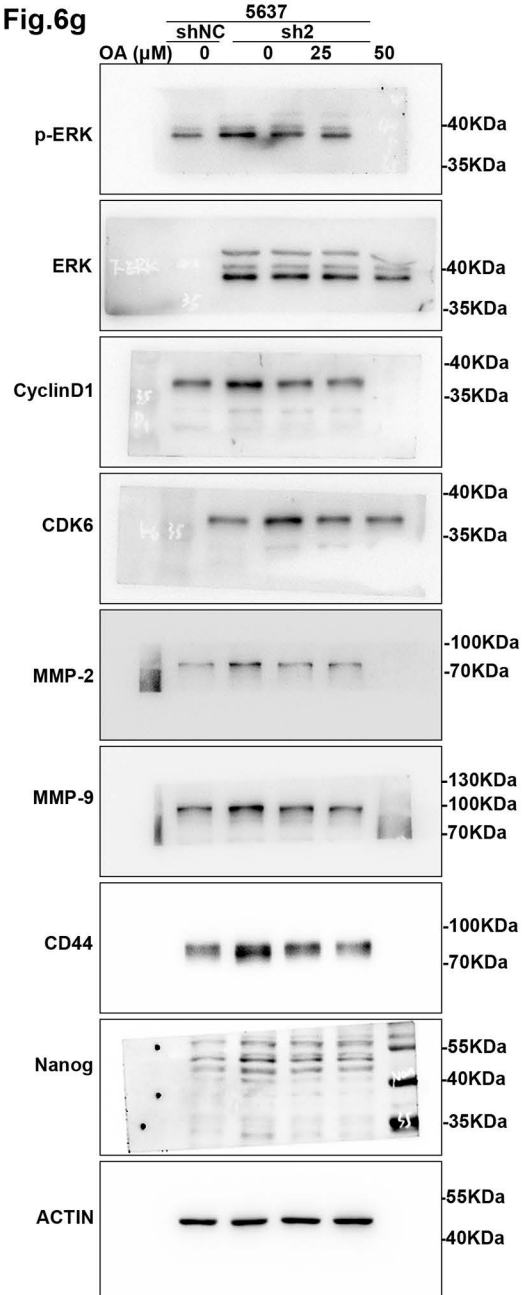

**Fig.7c**

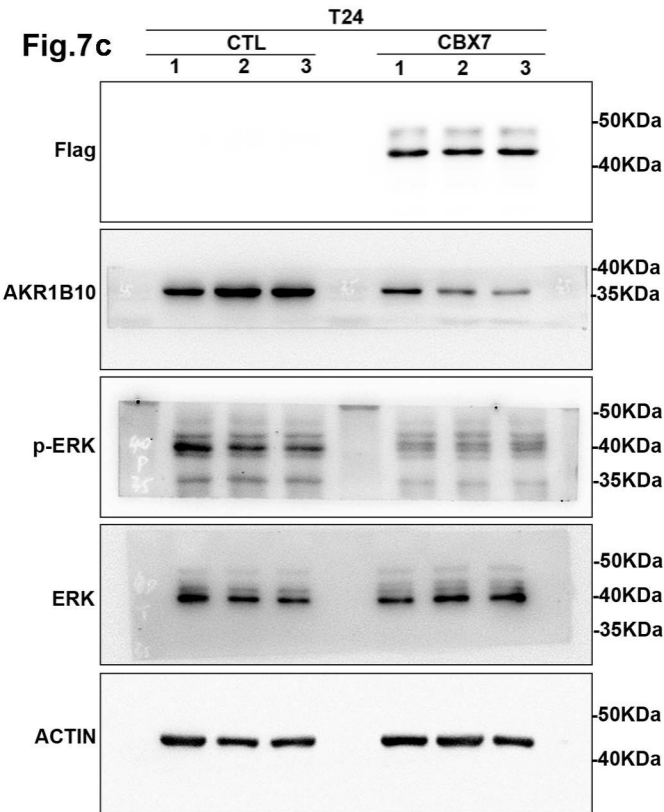

**Fig.S2a** UMUC-3  
CTL    CBX7

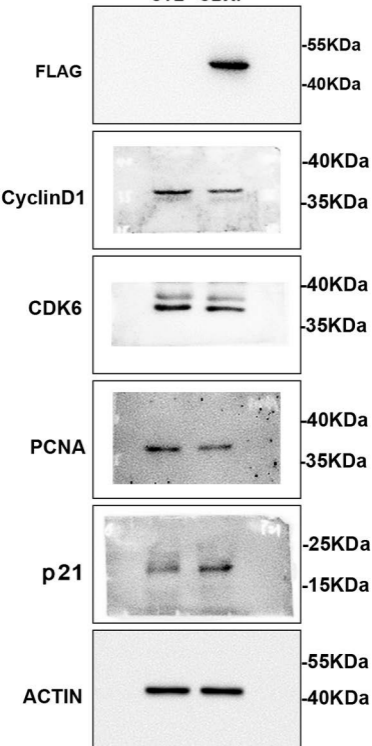

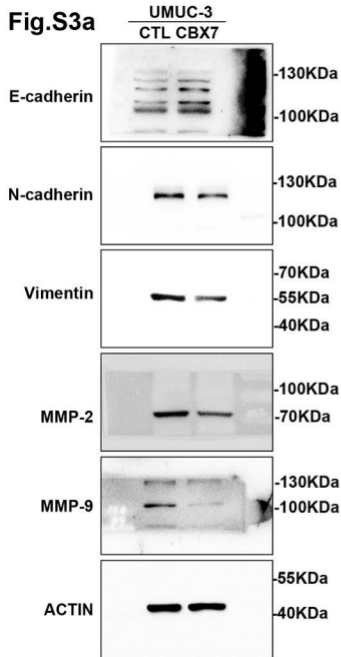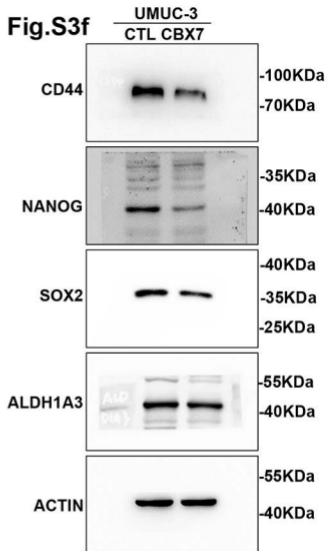

Supplement: Supplementary file 13 — Supplementary Figure 6 [file 41419_2021_3819_MOESM13_ESM.pdf]
